# Supplementary material for: The impact of music education on children’s cognitive and socioemotional development: A quasi-experimental study in the Guri Program in Brazil
Source: PLoS One. 2025 Oct 16;20(10):e0314355. doi: 10.1371/journal.pone.0314355 (PMC12530532; doi:10.1371/journal.pone.0314355)
Supplement: S1 File — The effects on brain Structures, social and cognitive skills in children exposed to the Guri Santa Marcelina program in Greater São Paulo: A quasi-experimental study. (PDF) [file pone.0314355.s002.pdf]

São Paulo State University
Institute of Arts
Júlio de Mesquita Filho
Music Department

Professor Graziela Bortz

The Effects on Brain Structures, Social and Cognitive Skills inChildren Exposed to the Guri Santa Marcelina Program in Greater São Paulo: A Quasi-Experimental Study

São Paulo
2018

The Effects on Social and Cognitive Skills in Children Exposed to the Guri Santa Marcelina Program in Greater São Paulo: A Quasi-Experimental Study

1. Summary

The question guiding this study is: how can participation in the Guri Santa Marcelina Program affect the development of social skills, cognitive abilities and brain structure in children aged between 6 and 7? Among the observational studies used in the health area, the *quasi-experimental* design verifies the causal relationship between exposure to a situation and the prognosis of an outcome. It has been widely used in research in situations where conducting a randomized control study would be unethical. This study will be used here for reasons inherent to the process of enrolling in the Program, prospectively throughout the 2020 school year, when data will be collected from the internal group (50 children) exposed to the Program and the control group (100 children, 50 of whom will be randomized to MRI), in order to measure, through this observation, the effects that the predictor (being exposed to the Program) has on the outcome variable (social and cognitive skills and changes in brain structure). Parents or guardians will sign the Informed Consent Form at the time of enrollment. The aims of this research are: 1) to evaluate the social effects of the Guri Santa Marcelina Program of the São Paulo State Department of Culture on children aged 6 and 7 in the communities served by the program using the ABEP economic classification criteria (2018), the SDQ questionnaire (Goodman & Goodman, 2011) of abilities and difficulties, the Questionnaire of Aggressive and Reactive Behaviors among Peers (Q-CARP; Borsa & Bandeira, 2014), verbal working memory (WISC-IV), concentrated, divided and sustained attention BPA (Rueda, 2013) and Raven's Colored Matrices test (Raven, Raven, & Court, 2003); 2) to observe possible structural changes in the brains of children exposed to the intervention of the Guri Santa Marcelina Program in 6 and 7 year olds by means of magnetic resonance imaging (MRI). Results of research conducted on programs inspired by the Venezuelan project based on orchestral music teaching known as *El Sistema* showed a significant improvement in students' academic performance when compared to the control group (Holochwost *et al.*, 2017b), cognitive (Habibi *et al.*, 2016), socio-emotional and musical (Ilari *et al.*, 2016). Similar results in a study conducted by Alemán *et al*. (2016) corroborate the behavioral benefits of children exposed to social vulnerabilities. In Brazil, although there are several social music education projects (Ação Social pela Música do Brasil - ASMB in Chapéu Mangueira, Alemão, Macacos, Cidade de Deus, Piraí, João Pessoa, Rondônia, Neojibá in Bahia, Projeto Guri in the interior and coast of São Paulo, Instituto Baccarelli, formalized in 1996 in the city of São Paulo, and Guri Santa Marcelina in Greater São Paulo), there are no quantitative studies or studies that have used imaging exams to measure the prognosis that exposure to social programs focused on music education presents as an outcome.

2. Introduction and justification

Neuroscientist Pinker (2015, p. 559) provocatively declared that music is "an auditory *cheescake*, an exquisite delicacy designed to delight the sensitive sites of at least six of our mental faculties", meaning that, although pleasant, musical manifestations are not essential for the preservation of human life. In response to Pinker, several scholars of musical cognition have taken a stand, including Huron (2001), saying that, although he is not convinced that music has evolutionary origins, he does not dismiss the possibility of investigation, and lists some assumptions that he thinks should be taken into account, such as: sexual partner selection for mating, as a behavior for conquest: "Darwin himself suggested that music may have arisen due to sexual selection in mate calling" (p. 61), social cohesion, effort to find a partner, and the need to find a partner. 61), social cohesion, cooperative effort, perceptual development, motor coordination development, conflict reduction, alertness and communication between generations. Trehub (2001) also studies the uniqueness of the baby's recognition of the mother's voice, and argues that this important connecting factor may have played a crucial role in the preservation of the species.

The artistic legacy left by *Homo sapiens* from the remote times of the Lascaut and Chauvet caves bears witness to the indescribable sensitivity of the first and only human to need to channel, through art, his acute perception of his own emotions and his surroundings with intense acuity and technical refinement. In the case of musical art, although it has been recognized throughout history by the great religions and political ideologies as an efficient social catalyst (Huron, 2001), its relevance still needs to be justified in the face of the utilitarian society of modern times.

As a result of the recent financial crisis in our country, and in response to the austerity measures recently imposed by the federal government, which have been extended to states and municipalities in recent years, we have seen exponential and growing cuts in government funding for the areas of Culture, Science and Education. Faced with such a crisis, it is crucial that the academic community reacts by using robust tools to rigorously measure the knowledge generated and its impact on social welfare and, consequently, so that the cultural goods generated by public projects and policies can be monitored and better understood. Although there is qualitative research that addresses the social benefits of projects involving music in Brazil (Weichselbaum & Nunes, 2016), no study has carried out extensive, quantitative research to assess the impact of such projects on society. This research aims to measure the impact that the Guri Santa Marcelina Program has on the communities around the centers where it operates in Greater São Paulo. To do this, the following tools will be used socioeconomic level according to the economic classification criteria of the Brazilian Association of Research Companies - ABEP (2018); SDQ questionnaire (Goodman & Goodman, 2011) which assesses four areas of difficulties (emotional symptoms, conduct problems, hyperactivity, relationship problems) and one area of ability (pro-social behavior); the Questionnaire of Aggressive and Reactive Behaviors among Peers (Q-CARP; Borsa & Bandeira, 2014), which assesses aggressive behavior and different forms of reaction to aggression by the child; verbal working memory (WISC-IV), concentrated, divided and sustained attention (Rueda, 2013) and the Raven Colored Matrices test (Raven, Raven, & Court, 2003), an instrument used to assess non-verbal intelligence, analogical reasoning and eductive ability in children aged 5 to 11. and magnetic resonance imaging (MRI) scans. Further details on the method of application, target audience and sample will be provided in the Methods section.

These data are important because we assume that they may reflect significant social changes caused by interventions aimed at involving children and young people in activities that promote their ability to concentrate, work collaboratively, develop convergent - linear - thinking and divergent - creative - thinking (Fink, Grabner, Benedek, & Neubauer, 2006) in unfavorable socio-economic environments, environments that do not offer opportunities for access to a balanced education, which will be discussed in section 2.2 (Effects of stress on the brain). Thus, it can be assumed that the use of the SDQ scale could measure the program's impact on changes in the ability to manage internal and social conflicts in these children who were or were not exposed to the intervention.

2.1 Musical abilities and the cerebral hemispheres

The fact that there are image records that show the activation of brain areas responsible entirely for sensory processes in the act of imagining and remembering alters the original conception of the separation between perception and memory, as well as that which

considered the areas responsible for certain processes to be watertight (Springer & Deutsch, 1998, p. 223). In an example of the complementarity between the hemispheres in complex tasks, Sergent , Zuck, Terriah and MacDonald (1992) found activation in the temporal lobes of both hemispheres when the research participants (musicians) listened to a piece of music, which did not occur when listening to a scale. Despite the obvious activation in both hemispheres in the auditory cortex, the latter hearing was able to activate only the left temporal lobe, which is surprising since it is known that the right temporal lobe is responsible for processing melodies and timbres.

Another interesting piece of evidence found by Sergent *et al*. (1992) is that the silent reading of sheet music activates regions that are not normally activated when reading words (the junction of the left occipital and parietal lobes), which, according to Springer and Deutsch (1998, p. 227) suggests that "the relevant information in musical notation is provided by the analysis of the spatial location of the notes on the staff (which is directly related to the intervals of the tones)". Furthermore, in the keyboard score reading task, two brain regions were activated: 1) the superior parietal lobe in both hemispheres, and 2) the left frontal lobe "immediately above Broca's area", which suggests proprioceptive processing[1] , which is characteristic of the right hemisphere, and analytical sequential processing of the left hemisphere. In addition, the researchers suggest that, due to its proximity to Broca's area, the motor control of playing the piano would activate an area that would be equivalent to speech, but related to the musical aspect. This study explains why musicians who suffer lesions in the left hemisphere (as in Ravel's case)[2] have some musical skills affected and others not.

> Sergent also suggested that these findings are consistent with both the modular view of brain organization, which emphasizes the unique competencies of specific brain regions, and the distributed view, which the multiple processes involved in musical performance and most other forms of human expression make necessary. (Springer & Deutsch, 1998, p. 228)

The complex mental activity of playing from a score on the piano requires multiple processes in different cortical regions. We don't know if it will be possible to identify structural changes in the brain in just one year of intervention by the Guri Santa Marcelina Program, which we will observe using MRI scans before exposure and at the end of the first school year.

2.2 Effects of stress on the brain

Children who grow up in unfavorable socio-economic environments, living in constant threat of violence to life, sexual violence, hunger, financial instability, lack of resources, housing, emotional support, education, health, protection, harassment and humiliation, are certainly vulnerable to emotional stress.

According to Juruena, Clearea and Pariante (2004, p. 190), the hypothalamic-pituitary-adrenal (HPA) endocrine axis "plays a fundamental role in the response to external and

---

[1] Proprioception belongs to the sensory modality of stimuli on the body, being a submodality that refers to the perception, conscious or not, of the static or dynamic position of the body (Lent, 2010, pp. 187-229).

[2] Ravel retained the ability to recognize melodies, detect errors in execution and differences in pitch, but could no longer classify musical notes or play or transcribe music. The case is reported in Springer and Deutsch (1998, p. 226).

internal stimuli, including psychological stressors". Dysregulation in the balance of this axis can be a normal adaptation and protection response of the organism to temporary stress, but it can make the hippocampus vulnerable to health damage described in the literature as psychiatric disorders (Juruena, Clearea & Pariante 2004; Stephens & Wand, 2012), low immunity (Corwin *et al.*, 2013) and pathophysiologies resulting from ongoing stress (Jelinek, Randjbar, Seifert, Kellner & Moritz, 2009; Zalewski, Lengua, Kiff & Fisher, 2012), and can even affect declarative memory and potentiate damage caused by ischemia and seizures (McEwen, 2001). "The detrimental actions of glucocorticoids in such conditions have been termed 'allostatic load', referring to the cost to the body of adapting to adverse conditions" (McEwen, 2001, p. 265).

McEwen (2001) also reports an increase in aggressive behavior and fear in laboratory rats subjected to continuous stress for 21 consecutive days. When discussing structural changes that occur in the brain caused by stress-related disorders, such as depression and Post-Traumatic Stress Disorder (PTSD), the author mentions alterations in the hippocampus, amygdala and prefrontal cortex. He adds that the effects of childhood deprivation continue throughout adulthood and lead to atrophy in the hippocampus and other brain structures. He concludes by warning that "early experience and a history of childhood abuse and neglect must be considered in relation to chronic pain and increased chemical sensitivities" (MacEwen, 2001, p. 272).

There are a number of health and developmental consequences for children of growing up in poverty, stress being just one of them (Blair, 2010; Blair, Berry, Mills-Koonce, Granger & the FLP Investigators, 2013; Sheridan *et al.*, 2012). However, it is worth warning, as it is a crucial problem due to the serious alterations in the neurological response system and its effects on the structural and functional development of the brain that "promote or prevent the development of reflective and guided self-regulation of behavior as that necessary for successful school performance" (Blair *et al*., 2013, p. 1). The author believes that this response should not be confused with the beneficial and adaptive response of the organism exposed to stress for a short period of time, since in this case it causes an overload that results in a series of clinical pathologies, psychopathologies, as well as delays in cognitive development and learning disorders.

Given this scenario, there is no denying that the chances of children and adolescents living under constant stress need support to enable them to deal with their development with the fluency that any citizen should be able to. In the US, expressions have recently emerged in education that refer to these issues, such as: *achievement gap* and *opportunity gap* (Milner, 2012), based on the understanding that opportunities are not equally distributed, recognizing that children who grow up in unfavorable socioeconomic environments tend to perpetuate the vicious circle of poverty.

2.3 Social and socio-educational projects in music

In 1975, the program known as *El Sistema* in Venezuela was conceived and founded by musician José Antonio Abreu, with the aim of "systematizing the instruction and collective and individual practice of music through symphony orchestras and choirs, as instruments of social

organization and humanistic development" (Ministerio del Poder Popular de Venezuela, 2018). On the path traced by this program, others followed in various parts of the world[3] . In Brazil, at least two projects mention inspiration from *El Sistema*: Ação Social pela Música do Brasil (ASMB), founded in 1994 by conductor David Machado, which has been implemented in various regions of the country, with four centers in the city of Rio de Janeiro (Chapéu Mangueira, Alemão, Macacos and Cidade de Deus), two in Petrópolis, one in Piraí, one in João Pessoa and another in Rondônia; and Neojibá, in Bahia, created in 2007 by conductor and pianist Ricardo Castro.

In the state of São Paulo, other social music education programs include the Guri Project, which covers the interior and coast of São Paulo, founded in 1995, the Baccarelli Institute, formalized in 1996, and the Guri Santa Marcelina Program, launched in 2008 in Greater São Paulo, which are not necessarily or directly influenced by the Venezuelan program. In addition to offering music training, they all aim to include children and teenagers at social risk.

The Guri Santa Marcelina Program has the following values:

> • Social responsibility
> • Using culture (music) as a link and tool for developing human values and promoting social inclusion
> • Qualification of teaching, welfare and administrative staff
> • Artistic excellence: quality of teaching materials and musical practice (facilities, methods and musical instruments)
> • Continuity, sense of process and social transformation
> • Commitment of students, families and communities to the program
> • Compliance with the precepts of the Statute of the Child and Adolescent (ECA)
>
> (Secretary of Culture of the State of São Paulo, SEC-SP, 2017)

As this is a public program run by the São Paulo State Government's Department of Culture, managed by the Santa Marcelina Social Organization, which has hired social workers on its staff, as it has a wide reach in underprivileged communities in Greater São Paulo, due to the quality of its facilities, the working conditions of its employees, the viability of the dialogue with its leaders, and the institutional support, this research project aims to examine the impact of the program on the communities it serves.

In the US, the initial report of the research conducted on the programs inspired by the Venezuelan *El Sistema* project conducted in that country states that the target audience of such projects usually has limited access to balanced education in their communities, and they hope, through the project, to "recognize and build children's and families' aspirations and wealth; buffer the effects of risks associated with poverty; demonstrate how engagement with musical learning fuels individual growth in other areas of development and learning" (Holochwost, Wolf, & Bose, 2017a). The preliminary results of the study conducted between 2014 and 2017 support that participation in the programs during these years showed significant results of improvement in the students' academic performance when compared to the control group (Holochwost *et al*., 2017b), cognitive (Habibi, Cahn, Damasio & Damasio,

---

[3] According to the MPP *website* (2018), there are 35 countries that have started to implement programs directly inspired by the Venezuelan *El Sistema*. Available at: http://fundamusical.org.ve/category/el-sistema/impacto-social/#.WoiNW-ZzLIU. Accessed on 17/02/2018.

2016), socio-emotional and musical (Ilari, Keller, Damasio & Habibi, 2016). Other important results observable in subgroups in this research refer to the fact that boys enrolled in the programs showed higher rates of perseverance than their non-enrolled peers, lower levels of aggression and higher levels of cooperation (remembering that aggression can be shown to be one of the side effects of stress on the brain). The report emphasizes that the result "is not automatic, but requires careful program design and implementation, as well as caution in the hiring of teachers and their follow-up," since there are differences in results from one program to another (Holochwost *et al*., 2017a).

Similar results were found in the study by Alemán *et al*. (2016) conducted in Venezuela itself, which found benefits from the point of view of overcoming behavioral difficulties, reducing aggression among peers, especially among subgroups of boys whose social vulnerabilities include excessive exposure to violence and children of mothers with lower levels of education.

These indices, measured on validated scales, are indicators that children exposed to socially vulnerable environments, and who would not be able to overcome the difficulties in their living environment without the intervention of public policies aimed at their inclusion, in just a few years of participating in socio-educational programs like *El Sistema*, or inspired by it, have been able to overcome emotional barriers that might seem insurmountable, as happens in environments surrounded by the vicious circle of poverty. We believe that the study of music can offer tools that help to activate the creative brain, convergent and divergent thinking, so that these children in situations of social vulnerability can build alternative paths to the poverty that has been destined for them, and so that it does not perpetuate itself in their lives and in future generations.

3. Objectives

a. To evaluate the cognitive and social effects of the Guri Santa Marcelina Program of the São Paulo State Department of Culture on children aged 6 and 7[4] in the communities served by the program in terms of behavioral and emotional problems assessed through questionnaires and tests.
b. To observe possible structural changes in the brains of children exposed to the Guri Santa Marcelina Program intervention in 6 and 7 year olds by means of magnetic resonance imaging (MRI).

4. Method

4.1 Research topic

The question guiding this study is: how can participation in the Guri Santa Marcelina Program (predictor X) interfere with the development of social skills, cognitive abilities and brain structure in children aged between 6 and 7 (outcomes Y)?

4.2 Research design

[4] This is the starting age range served by the Guri Santa Marcelina Program.

This is a longitudinal study with a *quasi-experimental* design. This design makes it possible to verify, like a randomized controlled trial, the effect of a given exposure (Guri project) to a situation and the prognosis of an outcome (our behavioral, cognitive and neuroimaging measures). However, some analytical and sampling precautions and the selection of *control* variables (see below for *control instruments)* are fundamental when planning the study, since the quasi-experiment does not have randomization, which is fundamental to avoid biases arising from confounders that could alter the chance of a given child participating in the Guri project or not (for more details, see the literature on causality and the principle of ignorability (Greenland & Mansournia, 2015). This type of design has been widely used in research in situations where conducting a randomized control study would be unethical or even impossible for logistical reasons, such as intentionally exposing people to risk factors (e.g. cigarettes and asbestos). Considering our context, it would not be possible, given the functioning and structure of the Program itself, to select a group of children who had never had music lessons and from this initial selection, randomly draw those who would participate in the Guri project and those who could not participate in the Guri project during the period of almost a year of study. We thought of proposing to Guri that after a year, the children who had been randomly selected for the control group would be put on a waiting list and would have the possibility of being guaranteed a place for the following year; however, such a procedure is neither acceptable nor ethical in view of Guri's structure. In short, we chose this mode of study, since it would be impossible, for ethical reasons inherent in the democratic process of enrolling in the Program[5] , to conduct a randomized study, which is the *gold-standard* design within classical biostatistics in the process of causal inference (Boutron, Altman, Moher, Schulz & Ravaud, 2017; Greenland & Mansournia, 2015).

4.3 Instruments

4.3.1 Control instruments

Questionnaire of economic classification criteria from the Brazilian Association of Research Companies - ABEP (2018). Created to assess socioeconomic status based on household samples, it contains 9 items that assess a) ownership of durable consumer goods; b) type of water supply system and street paving; c) number of residents in the household; d) family composition; e) level of education of the head of the household. The result of the questionnaire is a measure stratified into 5 socio-economic classes: A (subdivided into A1 and A2), B (also subdivided into B1 and B2), C, D and E.

Raven's Colored Progressive Matrices (CPM) - Special Scale (Raven *et al.*, 2003): an instrument used to assess non-verbal intelligence, analogical reasoning and eductive ability in children aged 5 to 11. It is one of the most widely used instruments for assessing non-verbal intelligence in children and adults (Lúcio *et al.*, 2017; Pasquali, Wechsler, & Bensusan, 2002). It has 36 items divided into three series with 12 items each, in gradual order of difficulty: series

[5] The program does not have a selection process. Although there is a waiting list, which could function as a control group in the randomized study, it would be unethical to force the children on this list to wait a whole year before they can enroll, since, due to dropouts, it is possible to get a place during the year.

A, Ab and B. Each item in the series is made up of a figure with a piece missing, and the child's task is to indicate the figure that completes the missing part within the 6 answer options presented. The maximum score for the task is 36 points and the intelligence classification is given in percentiles. A participant who scores in the 5th percentile (V classification) is at risk of intellectual difficulties and will therefore be excluded from the sample.

4.3.2 Test instruments

***Strengths* and *Difficulties* Questionnaire (SDQ)** is a validated measure, translated and culturally adapted into more than 15 languages, with references that help us to have internationally standardized measurement parameters. The questionnaire was originally developed by Goodman (1997), with the most recent version by Goodman and Goodman (2011), and aims to provide a screening of behaviors, emotions and relationships of children and adolescents. It has been validated and adapted for Brazilian Portuguese (Stivanin, Scheuer, & Assumpção, 2008; Woerner *et al*., 2004). The questionnaire has two versions, which can be answered by parents or teachers (the same items, adapted for the respondent). There are 25 statements that must be answered on a 3-point Likert scale, made up of the options: false, more or less true and true (for example: "are you kind to younger children?"). The 25 items are organized into 5 dimensions (emotional symptoms, conduct, hyperactivity, relationship problems and pro-social behaviour). Some items are reversed on the scale (e.g. in the hyperactivity section, if you answer "false" to the question "thinks before acting", you score 2 points; but if you answer "is constantly restless or agitated",you score zero points. This implies that the higher the score, the greater the difficulties. Similarly, in the case of strengths (sociability), they increase as the scores increase. The result of each scale can be evaluated if at least 3 items are scored. A score of 0 to 40 is given for difficulty items (sociability items are scored from 0 to 10). There is a second part containing impact information, or assessing stress and general impairment, which must be answered if the parent or teacher answers yes to the first question (if they answer negatively, they score zero and the application is stopped). If the impact supplement shows a score of 0, it is considered normal, 1 borderline and 2 abnormal.

Psychological Battery for the Assessment of Attention (BPA, Rueda, 2013) - this is a set of subtests that assess three domains of attention: concentrated attention (the ability to focus, select and maintain attention on target stimuli and ignore competing stimuli); divided attention (the ability to pay attention to more than one stimulus at the same time, responding to the different needs of the environment); and sustained attention (the ability to switch attention from one stimulus to another). The test presents validity and accuracy studies and norms for people between 6 and 82 years of age.

WISC-IV Digits Subtest (Wechsler, 2013): assesses verbal working memory and verbal working memory. Sets of numbers are presented to the children in ascending order (2 to 8 sequences) and the children must repeat them in the same order (direct digits) or in the reverse order (inverse digits) in which the numbers were presented. Each item has two attempts, and the subtest is interrupted when the child misses two attempts at the same item. The score for each attempt is one point. Thus, the maximum score in each subtest subdivision (direct or inverse) is 16 points, giving a total subtest score of 36 points.

Questionnaire on Aggressive and Reactive Peer Behavior (Q-CARP) - a questionnaire translated and adapted into Brazilian Portuguese by Borsa and Bandeira (2014). The questionnaire contains a total of 20 questions asked by the applicator to the child, involving two different scales: the Aggressive Behavior Scale - ACE, made up of five items that assess physical and verbal aggression (e.g. kicking or slapping) and three control items that are not scored and serve as a control (e.g. telling jokes). These items refer to the frequency of behaviors and are asked as "how often does it happen to you ", with each item having a 4-point Likert scale (every day, sometimes, infrequently and never). The Aggression Reaction Scale (ARS) consists of 12 phrases that represent different ways in which children commonly react to aggression from their peers. The question asked of these items is "when a classmate of yours" followed by the statements, which can signal aggressive reactions (e.g.: hits or pushes you, do you hit your classmate?) or other forms of reaction, such as seeking support (e.g.: hits or pushes you, do you tell the teacher?) and internalized emotional reactions (e.g.: hits or pushes you, do you cry and get punched?).

4.4 Data collection procedure

Among the children aged 6 and 7 who currently attend two hours of classes in the Guri Santa Marcelina Program, there are 653 enrolled in all the centers. The Program operates in 46 centers, each with different characteristics and peculiarities. This information will be analytically adjusted so that it is possible to verify differences in the influence of the socio-economic effect of the places where the Program centers are located.

In order to plan the logistics of applying the tests and imaging exams, a pilot test will be carried out from August to December 2019. Fifty parents/guardians of children who attend the Guri project will be approached to answer the SDQ in order to estimate the prevalence of children who participate in the project and who present the cut-off points for difficulties presented in the SDQ questionnaire. Participants who meet these cut-off points will undergo the planned individual assessments (psychological tests) in order to check the time needed to apply the instruments and whether there is a need to adjust the cut-off points (e.g. use borderline or abnormal). It will also serve to predict the expected proportion of children who will have to be excluded due to lower than expected intellectual performance (which will probably result in an increase in the number of children drawn to make up the EG for the individual assessments in order to obtain a sample size of 50 participants).

The main study will be carried out prospectively throughout the 2020 school year, when data will be collected from two groups: 1) the internal group, made up of 50 children aged 6 and 7 who have been exposed to the Program; and 2) the control group, made up of 100 children from the same age group and neighborhood who do not attend - and have never attended - the Program.[6] The groups will be assessed at two different times for the screening

[6] The 1:2 imbalance was done on purpose to help estimate the potential effects of the intervention in the statistical analysis process. The parameters for the sample calculation were: two-tailed t-distribution test for two independent samples with power (1-error type II) of 80%, significance level of 0.015 [data will be 4 main behavioral measures of a continuous nature as the outcome], allocation of 1:2. This design allows us to identify effects of moderate magnitude Cohen's d=0.58 (Cohen, 1988). In other words, we would be able to identify differences between the two groups, if they exist, if they are moderate. For more details on the formulas and effect sizes, see Cohen (1988), Julious (2010), Machin et al. (2009), Ryan (2013).

instruments and the cognitive/emotional tests: before the start of the school year (screening and pre-test) and at the end of the school year (post-test). The procedures for composing the groups (treatment and control) and for the evaluations are described below.

On the day the children enroll in the Guri project, the parents/guardians will be informed about the possibility of their children taking part in the research and will be asked to sign the Informed Consent Form (ICF). After signing, they will be asked to answer the SDQ questionnaire - version for parents and the ABEP questionnaire (2018) to assess socioeconomic status. This procedure constitutes the screening phase and aims to identify the children who will be attending Guri in 2020 and who have problems in the five areas assessed by the SDQ (namely emotional symptoms, conduct problems, hyperactivity, relationship problems). With the results of the screening, 50 children will be randomly selected[7] to take part in the individual research tests. Once the children who will make up the experimental group (EG) have been selected, the schools they regularly attend will be identified to make up the control group (CG). After authorization from the school management to take part in the research, the parents of the children of the same sex and who attend the same class as each child in the EG selected will be invited to take part in the research and will receive the ICF together with the SDQ and the ABEP questionnaire. Parents who return the signed ICF will have the SDQ corrected and participants with the same cut-off points as the children in the experimental group will be drawn to make up the CG.

Once the groups have been put together, the children will go for individual assessments (pre-test), in which their non-verbal intelligence (Raven's test) will be assessed (Raven *et al*., 2003).

The neuroimaging evaluations will be organized according to a specific schedule, since it requires participants to travel to the evaluation site. For the neuroimaging assessments, 50 children from the control group will be randomized to be sent for magnetic resonance imaging (MRI), in addition to the 50 who will be exposed to the Program[8] , under the supervision of researcher Prof. Dr. Andrea Jackowski from the Department of Psychiatry at Unifesp, a collaborator on this project.

After the end of the school year, the CG and SG children will be reassessed on all individual tasks, including imaging tests, in order to investigate the effect of the treatment on the dependent variables.

4.5 Collecting imaging tests

All the children underwent a desensitization protocol with a team of psychologists before the exam, in order to increase acceptance and reduce movement during the exam, since the entire image acquisition protocol lasts 20 minutes.

The images will be acquired at the Department of Diagnostic Imaging (UNIFESP) on a 3 Tesla device. All images will be analyzed at the Federal University of São Paulo (UNIFESP), at the Interdisciplinary Laboratory of Clinical Neurosciences (LiNC-UNIFESP), coordinated by Prof. Dr. Andrea Jackowski.

---

[7] This figure may vary upwards, depending on the results of the pilot study to assess intelligence.

[8] The Guri Santa Marcelina Program has a technical cooperation agreement with the Municipal Department of Education, which will facilitate access to the control group through the schools located near the centers where the Program operates.

The image acquisition protocol includes the following sequences:

- An exploratory series of sagittal images (nine to eleven 5 mm slices at 1 mm spacing).
- Structural neuroimaging: T1-weighted images will be acquired with the following acquisition parameters: MPRAGE; TR: 6.8; TE: 3.2; slice thickness: 1.2; Matrix 256 x 240; nutation angle: 9; voxel size: 1x1x1.2; number of slices: 150.
- Resting-state fMRI (functional magnetic resonance imaging): During the acquisition of this sequence, subjects will be asked to look at a fixed point without performing any specific task. The images will be acquired with the following acquisition pattern: EPI; TR: 2000; TE 30; voxel size: 3x3x3; acquisition matrix = 80x80 number of slices: 38
- Diffusion tensor imaging (DTI): Spin-echo single-shot echo planar imaging (EPI) will be acquired with the following parameters: TR 5800 ms; TE: 77 ms; matrix: 128 x 128; FOV: 256 x 256 mm; in-plane resolution 2 mm; ridge thickness: 3 mm, 15 directions, and b-value=0/1000.

4.6 Image Processing and Analysis

The MRI images will be processed using different platforms according to the imaging modality in question. T1 images will be processed using *FreeSurfer* software, which will be described in detail in the following subsections.

4.6.1 Structural measures

Measures of cortical thickness, local gyrification index and volume of cortical and subcortical structures will be calculated using the *FreeSurfer* software (surfer.nmr.mgh.harvard.edu) (Fischl et al., 1999; Dale et al., 1999]). *FreeSurfer* is a set of tools for post-processing and analyzing MRI data based on *watershed* algorithms and surface deformation models. *FreeSurfer* automatically segments cortical and subcortical structures, providing regional volume measurements by compartment (gray and white matter) as well as regional cortical thickness measurements. For this phase of the project, the procedure for longitudinal studies will be used, which increases the statistical power and reproducibility of the data. An average image and a specific template for each individual will be generated, using inverse registration. This approach allows for a significant increase in accuracy while maintaining the ability to detect anatomical changes [Reuter et al., 2012]. For the cortical thickness analyses, we will use the *SurfStat* package (http://www.math.mcgill.ca/keith/surfstat) for *Matlab*. This software allows longitudinal data to be analyzed using mixed-effects models. This procedure allows us to consider individuals with different scan intervals and data dependence for the same individual. The measurements of volume, average thickness, area and curvature of each region, generated during the segmentation and parceling stage, will be exported and used in analyses by region of interest (ROI), using the SPSS statistical package (Statistical Package for Social Sciences, version 20.0, Chicago IL, USA).

4.6.2 Resting-state fMRI

The functional magnetic resonance images (fMRI) will be pre-processed using routines from the FSL software (http://fsl.fmrib.ox.ac.uk/fsl) and AFNI (afni.nimh.nih.gov), encapsulated

in scripts based on the C-shell language. The pre-processing steps include: correction for head movement, removal of the skull and spikes, spatial smoothing using a Gaussian kernel (FWHM=8mm), band-pass temporal filtering (0.01-0.1Hz) with linear and quadratic detrending, realignment of the functional images with the structural images, where the latter are spatially normalized to the MNI152 template in a standard coordinate system, segmentation of the structural images into white matter, grey matter and cerebrospinal fluid, extraction of the average BOLD signal from the segmented areas, filtering of the BOLD signal from each region removing the effects of fluctuations in the cerebrospinal fluid, white matter, global signal, and the six movement parameters (rotation and translation) using multiple linear regression.

The inference of functional connectivity through the processing of fMRI data can be carried out in two ways: mapping from a seed region or analysis of regions of interest (ROI).Functional connectivity mapping via seed-voxel (consists of the following steps: 1) Data pre-processing; Step 2) Definition of a seed region; Step 3) Calculation of the correlation (2 to 2) between the BOLD signal of the seed region and each of the voxels in the image, using Pearson's or Spearman's Correlation coefficient; Step 4) Conversion of the correlation coefficient into a Z statistic, using Fisher's Z transform; Step 5) Thresholding of the map considering a certain level of significance (generally 5%, corrected by multiple comparisons using the FDR or FWE method). Thus, using the methodology described above, it is possible to identify which regions of the brain are functionally connected to the seed region. In intuitive terms, the aim is to find regions whose spontaneous activity (in a resting-state protocol) is associated with the activity of the seed region. These connectivity maps can then be used as features or input variables for machine learning and pattern recognition methods. A second complementary approach to functional connectivity mapping is based on the a priori definition of regions of interest (ROI; McIntosh, 1999). An ROI is defined by a set of spatially connected voxels representing a particular brain region (e.g. precuneus, dorsal portion of the anterior cingulum, etc.). The average BOLD signal between each of the voxels within the ROI is extracted and considered to be the representative signal for the region. This approach can be seen as a way of reducing the high dimensionality of the data, as the images are made up of thousands of voxels. Functional connectivity analysis is carried out considering a set of ROIs. In this way, it is possible to calculate the correlation coefficient (2 to 2 or partial) between the signs of the ROIs. ROIs can be defined by focusing on a specific circuit or on the whole brain using an atlas. For the exploratory approach using regions distributed throughout the brain, some pre-defined atlases can be used.

4.6.3 Diffusion Analysis

Post-processing of the diffusion-weighted images, resulting in diffusion tensor images (DTI), will be carried out using the FSL platform (free software), version 4.1.9 (Smith et al., 2006) according to the following steps:

- Correction of *eddy currents*, using the diffusion package (FDT) available in the FMRIB Library software (Smith et al., 2006);

- Extraction of the cranial box using the tool (BET) (Smith, 2006) with a variable extraction factor;

Using the FDT tool on the FSL platform and based on the tensors, the following maps will be constructed: fractional anisotropy (FA), whose normalized values vary between 0 and 1, will be inspected for any artifacts or residual movement, mean diffusivity (MD), and the eigenvalues λ1, λ2 and λ3. Depending on the type of study, it will be restricted to the use of FA maps or extended to the use of other maps.

The FA maps will be processed with *TBSS* (*Tract Based Spatial Statistics)* following the *pipeline* proposed by (Smith et al., 2006). As the sample is made up of children, it is considered more appropriate to perform subject-by-subject alignment in order to find the most representative individual in the sample. The selected target image will undergo a linear transformation followed by a translation (affine transformation) in the MNI152 standard space and each image in the study will be transformed into the MNI152 1x1x1 space by combining the non-linear transformation of all the images to the target image and, finally, the affine transformation of the target to the MNI152 space. The average of all these aligned FA images will result in a single 4D file with the average fractional anisotropy image. Based on this file, the group's average skeleton will be derived, and the most relevant tracts of each spatially normalized subject will be projected onto this skeleton. The recommended threshold of 0.2 will be applied. Based on the average skeleton (4D file) generated by TBSS, an automated extraction of regions of interest (ROI) will be carried out, based on the 20 tracts from the JHU (John Hopkins University) atlas of white matter tractography (Wakana et al., 2007; Hua et al., 2008). To extract the ROI for each subject, we used the *R-Project for Statistical Computing* program (version 3.0.2). In this way, the average value for each of the parameters separately (FA, MD, axial and radial diffusivities) will then be calculated based on the voxels belonging to the average skeleton. The values obtained will be extracted and processed in a statistical analysis appropriate to the design of each study using SPSS (Statistical Package for Social Sciences, version 20.0, Chicago IL, USA).

4.7 Methodological and ethical considerations

Since our hypothesis is that the Program's interference may be a predictor of changes in the prognosis of cognitive and social skills, and in brain structures as an outcome, we stress here the importance of observing, analyzing and interpreting these data so that, in the light of research that has shown the effects of social inequalities on the brain and health of children exposed to risk situations, we can provide a reading that allows society and those responsible for public policy projects to diagnose the latent benefits of their actions in the face of the evils of poverty.

It's important to note that, although there are several social music education projects (Ação Social pela Música do Brasil - ASMB in Chapéu Mangueira, Alemão, Macacos, Cidade de Deus, Piraí, João Pessoa and Rondônia, Neojibá in Bahia, Projeto Guri in the interior and coast of São Paulo, Instituto Baccarelli, formalized in 1996 in the city of São Paulo, and Guri Santa Marcelina in Greater São Paulo), there are no quantitative studies or studies that have used imaging exams to measure the prognosis that exposure to social programs focused on music education presents as an outcome in Brazil.

5. Form of Data Analysis

The data will be entered by the social workers and/or fellows/monitors into the *SurveyMonkey online* database, which will automatically generate Excel files and graphs, to be later statistically analyzed using Mplus 8 and Stata version 14.

For the analysis, mean, standard deviation, minimum and maximum values will be reported for each task used for the overall sample and by group, in the pre-test and post-test, as recommended by CONSORT, especially considering the extension to non-pharmacological studies (Boutron *et al*., 2017).

For inferential statistics, i.e. to verify the *generalizability* of the potential effects of the intervention versus the control group, the *Inverse Probability Weighting* technique will be used. This technique is suitable for different scenarios, such as studies with *missing data* in the different outcomes, *surveys* in which the subjects were not obtained through a random process and also for non-randomized studies comparing two interventions (Mansournia & Altman, 2016). Briefly, the statistical technique estimates the causal effect of the sample and removes possible confounders[9] , based on a logistic regression model that estimates the probability of exposure of a given person and uses the predicted probability as a weight in subsequent analyses (for more information see Cattaneo, 2010; Cerulli, 2014, 2015, Wooldridge, 2010).

The aim is to evaluate the dichotomous exposure of interest (participating in the Guri project *versus* not participating in the Guri project), assuming that exposure to the project will result in causal changes in the exposed group. In order to talk about causal effects without a random process, it is necessary to structure a Direct Acyclic Graph (DAG). From the DAG, which contains embedded non-parametric structural equations, it will be possible to check whether the target quantity of interest is identifiable from the variables collected by the researcher, applying, for example, the *back door criterion* of Pearl (2009a).

Confounders will be controlled by *matching*, based on the model that predicts similar levels of confounders between the exposure and non-exposure groups.

In addition to this central analysis, linear regressions will be carried out to evaluate the responses of the SDQ behavioral scale (Goodman & Goodman, 2011), the ABEP economic questionnaire (2018), the Questionnaire of Aggressive and Reactive Behaviors among Peers (Q-CARP; Borsa & Bandeira, 2014), verbal working memory (WISC-IV), concentrated, divided and sustained attention (Rueda, 2013) and the Raven's Colored Matrices test (Raven *et al*... 2003), 2003); the answers to which will be used as predictors of the outcome of exposure to the project.

6. Team

Responsible: Prof. Dr. Graziela Bortz

Previous research with Fapesp funding - Regular Aid: 2014/03322-1 (Productive and receptive melodic perception: evaluation and validation of criteria)

Result: Fapesp publication financial aid 2018/09500-0

Bortz, G., Germano, N. G., Cogo-Moreira, H. (2018). (Dis)agreement on sight-singing assessment of undergraduate musicians. *Frontiers in Psychology (Cognition).*

[9] Confounders are variables that can influence both the predictor and the outcome, causing spurious results. It is necessary to identify possible confounders and control them for more reliable results.

Collaborators:

Prof. Dr. Andrea Jackowski (Unifesp)
Prof. Dr. Patrícia Silva Lúcio (UEL)
Prof. Dr. Hugo Cogo-Moreira (Freie Universität Berlin/Unifesp)
Prof. Dr. Nayana Di Giuseppe Germano (UFSM)
Prof. Beatriz Ilari (USC, Thornton)

7. Implementation by the Partner Institution

The partner institution, Associação de Cultura, Educação e Assistência Social Santa Marcelina (Santa Marcelina Association of Culture, Education and Social Assistance), whose agreement with Universidade Estadual Paulista (Unesp) was signed on September 5, 2018, offers the collaboration of social workers, administrative technicians, interns, access to the centers where the Guri Santa Marcelina Program operates, as well as collaborating with the Municipal Department of Education, facilitating access to the students in the control group. Although there is no financial contribution, since it depends on government funding, the Association offers its institutional infrastructure.

The social workers of the Guri Santa Marcelina Program will be crucial players in the implementation of the work with the communities located in Greater São Paulo, as they are experienced professionals who enjoy the trust of these communities. These professionals and trainees from the partner institution will be trained together with the technical fellows to apply the questionnaires and scales, instruments that can be used for large-scale monitoring after the conclusion of the research proposed here, in the phase of implementing the results, being able to continue collecting data and applying the instruments capable of providing information and clues for constant monitoring of the evolution and intervention of the Program, both in each pole and in the Program as a whole.

8. Timetable

**Year**	**Jan-Mar**	**Apr-May**	**Jun-Jul**	**Aug**	**Sep-Oct**	**Nov**	**Dec**
**2019**				**PILOT: MRI, SDQ, ABEP, Raven and MT; BPA; Q-CARP;**		Evaluation of strategies and logistics	
**2020**	**SDQ, ABEP, Raven, MT; BPA; Q-CARP and** MRI	Survey Monkey	Partial report/ Accountability	**SDQ, ABEP, Raven, MT; BPA; Q-CARP and** MRI	Survey Monkey	**SDQ, ABEP, Raven, MT; BPA; Q-CARP and** MRI	Survey Monkey

**2021**	Statistical analysis<br>MRI analysis<br><br>Production of articles<br>Submission of results to international journals	Final Report / Accountability	Implementation Partner Institution
**2022**	Implementation Partner Institution		

9. References

Alemán, X., Duryea, S., Guerra, N. G., McEwan, P., Muñoz, J. R., Stampini, M., & Williamson, A. A. (2016). The effects of musical training on child development: a randomized trial of El Sistema in Venezuela. *Prevention Science, 18*, 865-878.

Brazilian Association of Research Companies - ABEP. (2018). Brazil economic classification criteria. Available at: http://www.abep.org/criterio-brasil Accessed on May 31, 2018.

Blair, C. (2010). Stress and the development of self-regulation in context. *Child Development Perspectives, 4*(3), 181-188.

Blair, C., Berry, D., Mills-Koonce, R., Granger, D., & the FLP Investigators (2013). Cumulative effects of early poverty on cortisol in young children: moderation by autonomic nervous system activity stress and poverty. *Psychoneuroendocrinology 38*(11), 2666-2675.

Borsa, J. C., & Bandeira, D. R. (2014). Cross-cultural adaptation of the questionnaire of aggressive and reactive behaviors among peers in Brazil. *PsicoUSF. 19*(2), 287-296.

Boutron, I. Altman, D. G., Moher, D., Schulz, K. F., & Ravaud, P. D. J. C. (2017) CONSORT Statement for Randomized Trials of Nonpharmacologic Treatments: A 2017 Update and a CONSORT Extension for Nonpharmacologic Trial Abstracts. *Annals of Internal Medicine. American College of Physicians, 167*(1):40. doi: 10.7326/M17-0046.

Cattaneo, M. D. (2010). Efficient semiparametric estimation of multi-valued treatment effects under ignorability. *Journal of Econometrics* 155: 138-154.

Cerulli, G. (2014). Ivtreatreg: A command for fitting binary treatment models with heterogeneous response to treatment and unobservable selection. *Stata Journal* 14: 453-480.

Cerulli, G. (2015). *Econometric Evaluation of Socio-Economic Programs: Theory and Applications*. Berlin: Springer.

Cohen, J. (1988). *Statistical Power Analysis for the Behavioral Sciences*. Hillsdale, NJ: Lawrence Erlbaum Associates.

Corwin, E. J., Guo, Y., Pajer, K., Lowe, N., McCarthy, D., Schmiege, S., Weber, M., Pace, T., & Stafford, B. (2013). Immune dysregulation and glucocorticoid resistance in minority and low income pregnant women. *Psychoneuroendocrinology 38*(9), 1786-1796.

Dale, A. M., Fischl, B., Sereno, M. I. (1999). Cortical surface-based analysis. I. Segmentation and surface reconstruction. *NeuroImage*, *9*(2):179-194.

Fink, A., Grabner, R. H., Benedek, M., & Neubauer, A. C. (2006). Divergent thinking training is related to frontal electroencephalogram alpha synchronization. *European Journal of Neuroscience*, *23,* 2241-2246.

Goodman, R. (1997). The Strengths and Difficulties Questionnaire: A research note. *Journal of Child Psychology and Psychiatry, 38*, 581-586.

Goodman, A., & Goodman, R. (2011). Population mean score predict child mental disorder rates: validating SDQ prevalence estimators in BritainStress. *Journal of Child Psychology and Psychiatry, 52*, 100-108.

Greenland, S., & Mansournia, M. A. (2015). Limitations of individual causal models, causal graphs, and ignorability assumptions, as illustrated by random confounding and design unfaithfulness. *European Journal of Epidemiology,30*(10), 1101-1110.

Habibi, A., Cahn, B. R., Damasio, A., & Damasio, H. (2016). Neural correlates of accelerated auditory processing in children engaged in music training. *Developmental Cognitive Neuroscience, 21*, 1-14.

Holochwost, S. J., Propper, C. B., Wolf, D. P., Willoughby, M. T., Fisher, K. R., Kolacz, J., Volpe, V. V., & Jaffee, S. R. (2017b). *Psychology of Aesthetics, Creativity, and the Arts, 11*, 147-166.

Holochwost, S. J., Wolf, D. P., & Bose, J. H. (2017a). Building strengths, buffering risk: evaluating the effects of El Sistema-inspired music programs in the United States. Available at: http://wolfbrown.com/images/books_reports/Building_Strengths_Buffering_Risk.pdf Accessed on 02/17/2018.

Hua, K., Zhang, J., Wakana, .S, Jiang, H., Li, X., Reich, D. S., Calabresi, P. A., Pekar, J. J., van Zijl, P. C., Mori, S. (2008). Tract probability maps in stereotaxic spaces: analyses of white matter anatomy and tract-specific quantification. *NeuroImage*, *39*(1):336-347.

Huron, D. (2001). Is Music an evolutionary adaptation? In I. Peretz & R. Zatorre (Eds.). *The Cognitive Neuroscience of Music* (pp. 57-75). Oxford: Oxford University Press.

Ilari, B. S., Keller, P., Damasio, H., & Habibi, A. (2016). The development of musical skills of underprivileged children over the course of 1 year: A study in the context of an El Sistema-inspired program. *Frontiers in Psychology*, 7.

Jelinek, L., Randjbar, S., Seifert, D., Kellner, M., & Moritz, S. (2009). The organization of autobiographical and nonautobiographical memory in posttraumatic Stress Disorder (PTSD). *Journal of Abnormal Psychology 118*(2), 288-298.

Julious, S. A. (2010). *Sample Sizes for Clinical Trials*. Boca Raton, FL: Chapman & Hall/CRC.

Juruena, M. F., Clearea, A. J., & Pariante, C. M. (2004). The hypothalamic-pituitary-adrenal axis, the function of glucocorticoid receptors and their importance in depression. *Revista Brasileira de Psquiatria, 26*(3), 189-201.

Lent, Robert (2010). *One Hundred Billion Neurons*. Rio de Janeiro: Atheneu.

Lúcio, P. S., Cogo-Moreira, H., Puglisi, M., Polanczyk, G. V., & Little, T. D. (2017). Psychometric Investigation of the Raven's Colored Progressive Matrices Test in a Sample of Preschool Children. *Assessment*. Advance online publication. doi:1073191117740205.

Machin, D., Campbell, M., Tan, B. T., Tan, S. H. (2009). *Sample Size Tables for Clinical Studies.* 3rd Edition. Hoboken, NJ: Wiley-Blackwell.

Mansournia, M. A, & Altman, D. G. (2016). Inverse probability weighting. *BMJ.* Research Methods & Reporting. doi: 10.1136/bmj.i189.

McEwen, B. S. (2001). Plasticity of the hippocampus: adaptation to chronic stress and allostatic load. *Annals of the New York Academy of Sciences*, 265-277.

McIntosh, A. R. (1999). Mapping cognition to the brain through neural interactions. *Memory*, *7*(5-6):523-548.

Milner, H. R. (2012). Beyond a test score: explaining opportunity gaps in educational practice. *Journal of Black Studies,*43(6), 693-718.

Ministerio del Poder Popular (MPP), Venezuela (2018). *The System.* Available at: http://fundamusical.org.ve/category/el-sistema/que-es-el-sistema/#.WoiI8uZzLIU Accessed on 17/02/2018

Pasquali, L., Wechsler, S., & Bensusan, E. (2002). Raven's Progressive Matrices for Children: a validation study for Brazil. *Avaliaçao Psicologica: Interamerican Journal of Psychological Assessment*, *1*(2), 95-110.

Pearl, J. (2009). Causal inference in statistics: An overview. *Statistics surveys*, *3*, 96-146.Pinker, S. (2015). *How the Mind Works* (3rd ed., L. T. Motta, Trad.). São Paulo: Companhia das Letras (Original work published in 1997).

Raven, J., Raven, J. C. & Court, J. H. (2003). Raven's Colored Progressive Matrices: Manual. (Trad. and Adapt. J. J. de Paula, C. G. M. F. Schlottfeldt, L. F. M. Diniz, G. A. A. Mizuta). São Paulo, SP: Pearson, 2018.

Reuter, M., Schmansky, N. J., Rosas, H. D., Fischl, B. (2012). Within-subject template estimation for unbiased longitudinal image analysis. *NeuroImage*, *61*(4):1402-1418.

Rueda, F. J. M. (2013). *BPA Collection - Psychological Battery of Attention*. São Paulo: Vetor.

São Paulo State Department of Culture (SEC-SP) (2017). *Guri Santa Marcelina Program.* Available at: http://gurisantamarcelina.org.br/estude-musica/sobre-o-programa/ accessed on 17/02/2018

Sergent, J., Zuck, E., Terriah, S., & MacDonald, B. (1992). Distributed neural network underlying musical sight-reading and keyboard performance. *Science, 257,* 106-109.

Sheridan, M. A., Foxd, N. A., Zeanahe, Charles H., McLaughlinb, K. A., & Charles A. Nelson (2012). Variation in neural development as a result of exposure to institutionalization early in childhood. *PNAS, 9*(23), 12927-12932.

Smith, S. M., Jenkinson, M., Johansen-Berg, H., Rueckert, D., Nichols, T. E., Mackay, C. E., Watkins, K. E, Ciccarelli, O., Cader, M. Z., Matthews, P. M. (2006). Tract-based spatial statistics: voxelwise analysis of multi-subject diffusion data. *NeuroImage*, *31*(4):1487-1505.

Springer, S., & Deutsch, G. (1998). *Left brain, right brain* (4th ed., T. Yoshiura). São Paulo: Summus Editorial (original work published in 1993).

Stephens, M. A. C., & Wand, G. (2012). Stress and the HPA axis role of glucocorticoids in alcohol dependence. *Alcohol Research: Current Reviews, 34*(4), 468-483.

Stivanin, L., Scheuer, C. I., & Assumpção Jr, F. B. (2008). SDQ (Strengths and Difficulties Questionnaire): identification of behavioral characteristics of child readers. *Psicologia: Teoria e Pesquisa*, *24*(4), 407-413.

Trehub, S. E. (2001). Musical predispositions in infancy: an update. In I. Peretz & R. Zatorre (Eds). *The Cognitive Neuroscience of Music* (pp. 3-20). Oxford: Oxford University Press.

Wakana, S., Caprihan, A., Panzenboeck, M. M., Fallon, J. H., Perry, M., Gollub, R. L., Hua K., Zhang J., Jiang, H., Dubey, P. (2007). Reproducibility of quantitative tractography methods applied to cerebral white matter. *NeuroImage, 36*(3):630-644.

Wechsler, D. (2013). *Weschsler Intelligence Scale for Children: WISC-IV. Technical Manual.* Translation of the original manual by Maria de Lourdes Duprat. (4. ed.). São Paulo: Casa do Psicólogo.

Weichselbaum, A., & Nunes, P. L. (2016). Contributions of music teaching in social projects: testimonials from graduates. *XVII ABEM Southern Regional Meeting,* 1-14.

Woerner, W., Fleitlich-Bilyk, B., Martinussen, R., Fletcher, J., Cucchiaro, G., Dalgalarrondo, P., ... & Tannock, R. (2004). The Strengths and Difficulties Questionnaire overseas: evaluations and applications of the SDQ beyond Europe. *European child & adolescent psychiatry*, *13*(2), ii47-ii54.

Wooldridge, J. M. (2010). *Econometric Analysis of Cross Section and Panel Data*. 2nd ed. Cambridge, MA: MIT Press.

Zalewski, M., Lengua L. J., Kiff C. J., & Fisher P. A. (2012). Understanding the relation of low income to HPA-axis functioning in preschool children: cumulative family risk and parenting as pathways to disruptions in cortisol. *Child Psychiatry Human Development 43*(6), 924-942.
