# Supplementary material for: The impact of music education on children’s cognitive and socioemotional development: A quasi-experimental study in the Guri Program in Brazil
Source: PLoS One. 2025 Oct 16;20(10):e0314355. doi: 10.1371/journal.pone.0314355 (PMC12530532; doi:10.1371/journal.pone.0314355)
Supplement: S2 File — Os efeitos sobre estruturas cerebrais, habilidades sociais e cognitivas em crianças expostas ao programa Guri Santa Marcelina na Grande São Paulo: Um estudo quasi-experimental. (PDF) [file pone.0314355.s003.pdf]

UNIVERSIDADE ESTADUAL PAULISTA
“JÚLIO DE MESQUITA FILHO”
INSTITUTO DE ARTES
Departamento de Música

PROFA. DRA. GRAZIELA BORTZ

Os Efeitos sobre Estruturas Cerebrais, Habilidades Sociais e Cognitivas em Crianças Expostas ao Programa Guri Santa Marcelina na Grande São Paulo: Um Estudo Quase-Experimental

São Paulo
2018

Os Efeitos sobre Habilidades Sociais e Cognitivas em Crianças Expostas ao Programa Guri Santa Marcelina na Grande São Paulo: Um Estudo Quase-Experimental

1. Resumo

A pergunta que norteia este trabalho é: de que modo a participação no Programa Guri Santa Marcelina pode interferir no desenvolvimento de habilidades sociais, nas habilidades cognitivas e na estrutura cerebral de crianças entre 6 e 7 anos? Dentre os estudos observacionais utilizados na área de saúde, o desenho quase-*experimental* verifica a relação causal entre a exposição a uma situação e o prognóstico de um desfecho. Tem sido largamente utilizado em pesquisas nas situações em que conduzir um estudo de controle randomizado seria antiético. Tal estudo será utilizado aqui por questões inerentes ao processo de inscrição no Programa, prospectivamente ao longo do ano escolar de 2020, quando serão levantados os dados do grupo interno (50 crianças) exposto ao Programa e do grupo de controle (100 crianças, das quais 50 randomizadas para MRI), para medir, por meio dessa observação, os efeitos que o preditor (estar exposto ao Programa) ocasiona sobre a variável de desfecho (habilidades sociais, cognitivas e mudanças na estrutura cerebral). Os pais ou responsáveis assinarão o Termo de Consentimento Livre e Esclarecido no momento da matrícula. São objetivos desta pesquisa: 1) avaliar os efeitos sociais do Programa Guri Santa Marcelina da Secretaria da Cultura do Estado de São Paulo em crianças de 6 e 7 anos nas comunidades atendidas pelo programa por meio do critérios de classificação econômica da ABEP (2018), o questionário SDQ (Goodman & Goodman, 2011) de capacidades e dificuldades, o Questionário de Comportamentos Agressivos e Reativos entre Pares (Q-CARP; Borsa & Bandeira, 2014), memória de trabalho verbal (WISC-IV), atenção concentrada, dividida e sustentada BPA (Rueda, 2013) e o teste de Matrizes Coloridas de Raven (Raven, Raven, & Court, 2003); 2) observar possíveis mudanças estruturais no cérebro de crianças expostas à intervenção do Programa Guri Santa Marcelina em crianças de 6 e 7 anos por meio de ressonância magnética (MRI). Resultados da pesquisa conduzida sobre programas inspirados no projeto venezuelano baseado em ensino de música orquestral conhecido como *El Sistema* apresentaram melhora significativa no desempenho acadêmico dos estudantes quando comparados ao grupo de controle (Holochwost *et al*., 2017b), cognitivo (Habibi *et al*., 2016), sócio emocional e musical (Ilari *et al*., 2016). Resultados similares em estudo conduzido por Alemán *et al*. (2016) corroboram os benefícios comportamentais de crianças expostas vulnerabilidades sociais. No Brasil, embora haja vários projetos sociais de educação musical (Ação Social pela Música do Brasil – ASMB em Chapéu Mangueira, Alemão, Macacos, Cidade de Deus, Piraí, João Pessoa, Rondônia, Neojibá, na Bahia, Projeto Guri, no interior e litoral paulista, Instituto Baccarelli, formalizado em 1996 na cidade de São Paulo, e Guri Santa Marcelina na Grande São Paulo), inexistem estudos quantitativos ou que tenham utilizado exames de imagem para medir o prognóstico que a exposição a programas sociais com foco em educação musical apresenta como desfecho.

2. Introdução e justificativa

O neurocientista Pinker (2015, p. 559) declarou provocativamente que música é "um *cheescake* auditivo, uma primorosa iguaria elaborada para deliciar os locais sensíveis de pelo menos seis de nossas faculdades mentais", querendo dizer com isso que, embora agradáveis, manifestações musicais não são essenciais para a preservação da vida humana. Em resposta a Pinker, vários estudiosos da cognição musical se posicionaram, entre eles Huron (2001), dizendo que, embora não esteja convencido de que a música tenha origens evolutivas, não dispensa a possibilidade da investigação, e elenca algumas suposições que, pensa, devam ser levadas em consideração, tais como: seleção de parceiro sexual para acasalamento, como um comportamento para conquista: "O próprio Darwin sugeriu que música pode ter surgido devido à seleção sexual em chamadas de parceiros" (p. 61), coesão social, esforço de cooperação, desenvolvimento perceptivo, desenvolvimento de coordenação motora, redução de conflitos, alerta e comunicação entre gerações. Trehub (2001) estuda, ainda, a singularidade do reconhecimento da voz da mãe pelo bebê, e argumenta que esse importante fator de conexão talvez tenha exercido papel crucial na preservação da espécie.

O legado artístico deixado pelo *Homo sapiens* dos tempos remotos das cavernas de Lascaut e Chauvet é testemunha da indescritível sensibilidade do primeiro e único humano a necessitar canalizar, através da arte, sua aguda percepção das próprias emoções e do ambiente que o circundava com intensa acuidade e refinamento técnico. No caso da arte musical, embora venha sendo reconhecida ao longo da história pelas grandes religiões e ideologias políticas como eficiente catalizador social (Huron, 2001), sua relevância ainda precisa se justificar diante da sociedade utilitária dos tempos modernos.

Em decorrência da crise financeira recente em nosso país, e como resposta às medidas de austeridade recentemente impostas pelo governo federal, que se tem estendido aos estados e municípios nos últimos anos, temos assistido a cortes exponenciais e crescentes de verbas governamentais destinadas às áreas de Cultura, Ciência e Educação. Diante de tal crise, é crucial que a comunidade acadêmica reaja utilizando ferramentas robustas que permitam medir com rigor o conhecimento gerado e o impacto deste no bem estar social e, por consequência, para que os bens culturais gerados pelos projetos e políticas públicas possam ser acompanhados e melhor compreendidos. Embora existam pesquisas qualitativas que abordem os benefícios sociais de projetos que envolvem música no Brasil (Weichselbaum & Nunes, 2016), nenhum estudo realizou pesquisa ampla e quantitativa para avaliar o impacto de tais projetos na sociedade. Esta pesquisa se propõe a medir o impacto que o Programa Guri Santa Marcelina exerce nas comunidades em torno dos polos em que atua na Grande São Paulo. Para isso, serão utilizadas as seguintes ferramentas: nível socioeconômico pelos critérios de classificação econômica da Associação Brasileira de Empresas de Pesquisa – ABEP (2018); questionário SDQ (Goodman & Goodman, 2011) que avalia quatro áreas de dificuldades (sintomas emocionais, problemas de conduta, hiperatividade, problemas de relacionamento) e uma área de habilidade (comportamento pró-social); o Questionário de Comportamentos Agressivos e Reativos entre Pares (Q-CARP; Borsa & Bandeira, 2014), que avalia comportamentos agressivos e diferentes formas de reação à agressão pela criança; a memória de trabalho verbal (WISC-IV), atenção concentrada, dividida e sustentada (Rueda, 2013) e o teste de Matrizes Coloridas de Raven (Raven, Raven, & Court, 2003), instrumento utilizado para avaliar a inteligência não-verbal, o raciocínio analógico e habilidade edutiva de crianças de 5 a 11 anos. e exames de ressonância magnética (MRI). Maiores detalhes sobre a forma de aplicação, público alvo e amostragem serão fornecidos no item Métodos.

Tais dados são importantes porque pressupomos que possam refletir mudanças sociais significativas causadas por intervenções que visem o envolvimento das crianças e jovens em atividades que promovam sua capacidade de concentração, trabalho colaborativo, desenvolvimento do pensamento convergente – linear, e do pensamento divergente – criativo (Fink, Grabner, Benedek, & Neubauer, 2006) em ambientes socioeconômicos desfavoráveis, ambientes esses que não ofereceriam oportunidades de acesso à educação balanceada, o que será discutido no item 2.2 (Efeitos do estresse no cérebro). Assim, pode-se supor que o uso da escala SDQ poderá aferir reflexos do programa em mudanças na capacidade de gerir conflitos internos e sociais dessas crianças que estiveram ou não expostas à intervenção.

2.1 Habilidades musicais e os hemisférios cerebrais

O fato de haver registros de imagem que verificam a ativação de áreas cerebrais responsáveis inteiramente por processos sensoriais no ato de se imaginar e de se lembrar

altera a concepção original de separação entre percepção e memória, assim como aquela que considerava que as áreas responsáveis por determinados processos seriam estanques (Springer & Deutsch, 1998, p. 223). Em um exemplo da complementaridade entre os hemisférios em tarefas complexas, Sergent, Zuck, Terriah e MacDonald (1992) encontraram ativações nos lobos temporais de ambos os hemisférios quando os participantes da pesquisa (músicos) ouviam uma peça musical, o que não ocorreu na audição de uma escala. A despeito da ativação óbvia em ambos os hemisférios no córtex auditivo, esta última audição foi capaz de ativar apenas o lobo temporal esquerdo, o que surpreende, já que é sabido que o lobo temporal direito é responsável pelo processamento de melodias e timbres.

Outra evidência interessante encontrada por Sergent *et al.* (1992) é que a leitura silenciosa de partituras ativa regiões que não são normalmente ativadas na leitura de palavras (a junção dos lobos occipital e parietal esquerdos), o que, segundo Springer e Deutsch (1998, p. 227) sugere que "a informação relevante na notação musical é proporcionada pela análise da localização espacial das notas na pauta (que é diretamente relacionada com os intervalos dos tons)." Ainda, na tarefa de leitura da partitura ao teclado, duas regiões cerebrais foram ativadas: 1) o lobo parietal superior em ambos os hemisférios, e 2) o lobo frontal esquerdo "imediatamente acima da área de Broca", o que sugere um processamento proprioceptivo[1], que é característico do hemisfério direito, e um processamento sequencial analítico do hemisfério esquerdo. Além disso, os pesquisadores sugerem que, pela proximidade à área de Broca, o controle motor do toque ao piano ativaria uma área que seria a equivalente à da fala, mas relativa ao aspecto musical. Esse estudo explica o porquê de músicos que sofrem lesões no hemisfério esquerdo (como o caso de Ravel)[2] têm algumas habilidades musicais afetadas e outras não.

> Sergent também sugeriu que estas descobertas são coerentes, tanto com a visão modular da organização do cérebro, que enfatiza competências únicas de regiões cerebrais específicas, como com a visão distribuída, que os múltiplos processos envolvidos na execução musical e na maior parte de outras formas de expressão humanas tornam necessária. (Springer & Deutsch, 1998, p. 228)

A atividade mental complexa de se tocar a partir de uma partitura ao piano exige múltiplos processamentos requisitados em diferentes regiões corticais. Não sabemos se em apenas um ano de intervenção do Programa Guri Santa Marcelina será possível identificar mudanças estruturais no cérebro, o que observaremos por meio de MRI antes da exposição e ao final do primeiro ano letivo.

2.2 Efeitos do estresse no cérebro

Crianças que crescem em ambientes socioeconômicos desfavoráveis, vivendo em constate ameaça de violência à vida, violência sexual, fome, instabilidades financeiras, falta de

---

[1] A propriocepção pertence à modalidade sensorial de estímulos sobre o corpo, sendo uma submodalidade que se refere à percepção, consciente ou não, da posição estática ou dinâmica do corpo (Lent, 2010, pp. 187-229).

[2] Ravel manteve a capacidade de reconhecer melodias, detectar erros de execução e diferenças de afinação, mas não conseguia mais classificar as notas musicais nem tocar ou transcrever músicas. O caso é relatado em Springer e Deutsch (1998, p. 226).

recursos, moradia, suporte emocional, educação, saúde, proteção, assédios e humilhações, são certamente vulneráveis ao estresse emocional.

De acordo com Juruena, Clearea e Pariante (2004, p. 190), o eixo endócrino hipotálamo-pituitária-adrenal (HPA) "exerce um papel fundamental na resposta aos estímulos externos e internos, incluindo os estressores psicológicos". A desregulação no equilíbrio desse eixo pode ser uma reposta normal de adaptação e proteção do organismo ao estresse temporário, mas pode tornar o hipocampo vulnerável a prejuízos à saúde descritos na literatura de transtornos psiquiátricos (Juruena,Clearea & Pariante 2004; Stephens & Wand, 2012), baixa imunidade (Corwin *et al.*, 2013) e fisiopatologias decorrentes de estresse continuado (Jelinek, Randjbar, Seifert, Kellner & Moritz, 2009; Zalewski, Lengua, Kiff & Fisher, 2012), podendo inclusive afetar a memória declarativa e potencializar danos causados por isquemias e convulsões (McEwen, 2001). "As ações prejudiciais dos glicocorticoides em tais condições foram denominadas 'carga alostática', referente ao custo para o corpo de adaptação a condições adversas" (McEwen, 2001, p. 265).

McEwen (2001) reporta ainda o aumento do comportamento agressivo e temor em ratos de laboratório sujeitos ao estresse contínuo durante 21 dias consecutivos. Ao discutir mudanças estruturais que ocorrem no cérebro causadas por desordens decorrentes de estresse, tais como depressão e Transtorno do Estresse Pós-Traumático (TEPT), o autor menciona alterações no hipocampo, amídala e córtex pré-frontal. Acrescenta que os efeitos de privações na infância acompanham toda a vida adulta, e levam a atrofias no hipocampo e outras estruturas cerebrais. Conclui advertindo que "a experiência inicial e uma história de abuso e negligência na infância devem ser consideradas em relação à dor crônica e incremento das sensibilidades químicas" (MacEwen, 2001, p. 272).

Há uma série de consequências à saúde e ao desenvolvimento de crianças derivada do crescimento em ambiente de pobreza, sendo o estresse apenas uma dentre elas (Blair, 2010; Blair, Berry, Mills-Koonce, Granger & the FLP Investigators, 2013; Sheridan *et al.*, 2012). No entanto, é digno de alerta, uma vez que se mostra um problema crucial devido às sérias alterações no sistema de resposta neurológica e seus efeitos no desenvolvimento estrutural e funcional do cérebro que "promovem ou impedem o desenvolvimento da autorregulação reflexiva e orientada do comportamento como aquela necessária para o êxito no desempenho escolar" (Blair *et al.*, 2013, p. 1). O autor avalia que não se deve confundir tal resposta com aquela benéfica e adaptativa do organismo exposto ao estresse por um curto espaço de tempo, uma vez que, nesse caso, causa uma sobrecarga que resulta numa série de patologias clínicas, psicopatologias, além de atrasos no desenvolvimento cognitivo e transtornos de aprendizagem.

Diante desse cenário, não se pode negar que as chances de crianças e adolescentes que vivem em constante estresse necessitam de suporte para poderem se capacitar para lidar com seu desenvolvimento com a fluência que qualquer cidadão deveria ter condições. Nos EUA, surgiram recentemente expressões na educação que se referem a essas questões, tais como: *achievement gap* e *opportunity gap* (Milner, 2012) a partir do entendimento de que as oportunidades não são igualmente distribuídas, reconhecendo que crianças que crescem em ambientes socioeconômicos desfavoráveis tendem a perpetuar o círculo vicioso da pobreza.

2.3 Projetos sociais e sócio-educacionais em música

Em 1975, o programa conhecido como *El Sistema* na Venezuela foi idealizado e fundado pelo músico José Antonio Abreu, com o objetivo de "sistematizar a instrução e a prática coletiva e individual da música através de orquestras sinfônicas e coros, como instrumentos de organização social e de desenvolvimento humanístico" (Ministerio del Poder Popular de Venezuela, 2018). No caminho traçado por esse programa, outros se seguiram em várias partes do mundo[3]. No Brasil, ao menos dois projetos mencionam a inspiração no *El Sistema*: a Ação Social pela Música do Brasil (ASMB), fundada em 1994 pelo maestro David Machado, implantado em várias regiões do país, tendo quatro núcleos na cidade do Rio de Janeiro (Chapéu Mangueira, Alemão, Macacos e Cidade de Deus), dois em Petrópolis, um em Piraí, um em João Pessoa e outro em Rondônia; e o Neojibá, na Bahia, criado em 2007 pelo maestro e pianista Ricardo Castro.

No Estado de São Paulo, entre outros programas sociais de educação musical, estão o Projeto Guri, que abarca o interior e litoral paulista, fundado em 1995, o Instituto Baccarelli, formalizado em 1996, e o Programa Guri Santa Marcelina, lançado em 2008 na Grande São Paulo, e que não são necessária ou diretamente influenciados pelo programa venezuelano. Todos têm como objetivo, além de oferecer formação em música, a inclusão de crianças e adolescentes em situação de risco social.

O Programa Guri Santa Marcelina apresenta como valores almejados:

> - Responsabilidade social
> - Utilização da cultura (música) como elo e ferramenta para o desenvolvimento de valores humanos e a promoção da inclusão social
> - Qualificação do corpo docente, de assistência social e administrativo
> - Excelência artística: qualidade do material de ensino e prática musical (instalações, métodos e instrumentos musicais)
> - Continuidade, senso de processo e transformação social
> - Comprometimento dos alunos, famílias e comunidades com o programa
> - Consonância com os preceitos do Estatuto da Criança e do Adolescente (ECA)
>
> (Secretaria da Cultura do Estado de São Paulo, SEC-SP, 2017)

Por ser um programa público da Secretaria da Cultura do Governo do Estado de São Paulo, gerido pela Organização Social Santa Marcelina, que conta com a participação de assistentes sociais contratados em seu quadro de colaboradores, por ter grande alcance em comunidades carentes na Grande São Paulo, pela qualidade de suas instalações, condições de contrato de trabalho de seus colaboradores, pela viabilidade na interlocução com seus dirigentes, e pelo suporte institucional, este projeto de pesquisa se propõe a examinar o impacto do programa nas comunidades por ele atendidas.

---

[3] Segundo o *site* do MPP (2018), existem 35 países que passaram a implantar programas inspirados diretamente no *El Sistema* venezuelano. Disponível em: http://fundamusical.org.ve/category/el-sistema/impacto-social/#.WoiNW-ZzLIU. Acesso em 17/02/2018.

Nos EUA, o relatório inicial da pesquisa conduzida sobre os programas inspirados no projeto venezuelano *El Sistema* conduzidos naquele país afirma que o público-alvo de tais projetos normalmente tem acesso limitado à educação balanceada em suas comunidades, e esperam, por meio do projeto, "reconhecer e construir aspirações e riquezas das crianças e das famílias; amortecer os efeitos dos riscos associados à pobreza; demonstrar como o engajamento com o aprendizado musical abastece o crescimento individual em outras áreas de desenvolvimento e aprendizado" (Holochwost, Wolf, & Bose, 2017a). Os resultados preliminares do estudo conduzido entre 2014 e 2017 sustentam que a participação nos programas durante esses anos mostrou resultados significativos de melhora no desempenho acadêmico dos estudantes quando comparados ao grupo de controle (Holochwost *et al*., 2017b), cognitivo (Habibi, Cahn, Damasio & Damasio, 2016), sócio emocional e musical (Ilari, Keller, Damasio & Habibi, 2016). Outros resultados importantes observáveis em subgrupos nessa pesquisa referem-se ao fato de que rapazes matriculados nos programas mostraram índices de perseverança maiores que seus pares não matriculados, menores níveis de agressividade e maiores níveis de cooperação (lembrando que agressividade pode se mostrar como um dos efeitos colaterais do estresse no cérebro). O relatório enfatiza que o resultado "não é automático, mas requer um cuidadoso desenho do programa e implantação, assim como cautela na contratação dos professores e seu acompanhamento", uma vez que há diferenças de resultado de um programa a outro (Holochwost *et al*., 2017a).

Resultados similares foram encontrados no estudo de Alemán *et al*. (2016) conduzido na própria Venezuela, que encontrou benefícios do ponto de vista de superação de dificuldades comportamentais, diminuição da agressividade entre os pares, especialmente entre os subgrupos de garotos cujas vulnerabilidades sociais incluem excessiva exposição à violência e filhos de mães com menor grau de instrução.

Tais índices medidos em escalas validadas são indicadores de que crianças expostas a ambientes de vulnerabilidade social, e que não teriam como superar as dificuldades em seu ambiente vital sem a intervenção de políticas públicas voltadas para sua inclusão, em poucos anos de participação nos programas sócio-educacionais como *El Sistema*, ou nele inspirados, foram capazes de ultrapassar barreiras emocionais que poderiam parecer intransponíveis, como o que sucede em ambientes cercados pelo círculo vicioso de pobreza. Acreditamos que o estudo da música possa oferecer ferramentas que colaborem para ativar o cérebro criativo, o pensamento convergente e divergente de maneira que essas crianças em situações de vulnerabilidade social possam construir caminhos alternativos à pobreza que lhes tem sido destinada, e para que esta não se perpetue em suas vidas e nas futuras gerações.

3. Objetivos

a. Avaliar os efeitos cognitivos e sociais do Programa Guri Santa Marcelina da Secretaria da Cultura do Estado de São Paulo em crianças de 6 e 7 anos[4] nas comunidades atendidas pelo programa em problemas de comportamento e emocionais avaliados por meio de questionários e testes.

[4] Esta é a faixa de idade inicial atendida pelo Programa Guri Santa Marcelina.

b. Observar possíveis mudanças estruturais no cérebro de crianças expostas à intervenção do Programa Guri Santa Marcelina em crianças de 6 e 7 anos por meio de ressonância magnética (MRI).

4. Método

4.1 Tema de pesquisa

A pergunta que norteia este trabalho é: de que modo a participação no Programa Guri Santa Marcelina (preditor X) pode interferir no desenvolvimento de habilidades sociais, nas habilidades cognitivas e na estrutura cerebral das crianças entre 6 e 7 anos (desfechos Y)?

4.2 Desenho da pesquisa

Trata-se de um estudo longitudinal com delineamento *quase-experimental*. Tal delineamento, permite verificar, tal como um ensaio controlado randomizado, o efeito de uma dada exposição (projeto Guri) a uma situação e o prognóstico de um desfecho (nossas medidas comportamentais, cognitivas e de neuroimagem). Porém, alguns cuidados analíticos, amostrais e seleção de variáveis de controle (ver abaixo *instrumentos de contole)* são fundamentais durante o planejamento do estudo, já que o quase-experimento não possui randomização que é uma peça fundamental para evitar vieses advindos de confundidores que poderiam alterar a chance de participação de uma dada criança no projeto Guri ou não (para maiores detalhes na literatura sobre causalidade e o princípio da ignorabilidade (Greenland & Mansournia, 2015). Esse tipo de delineamento tem sido largamente utilizado em pesquisas nas situações em que conduzir um estudo de controle randomizado seria antiético ou mesmo impossível por questões logísticas, como por exemplo, ao expor intencionalmente pessoas a fatores considerados de risco (e.g., cigarro e asbestos). Considerando o nosso contexto, não seria possível, dado o funcionamento e estrutura do próprio Programa, selecionar um conjunto de crianças que nunca tiveram aulas de música e a partir dessa seleção inicial, sortear, randomicamente, aquelas que participariam do projeto Guri e aquelas que não poderiam participar do projeto Guri durante o período de quase um ano de estudo. Pensou-se em propor para o Guri que após um ano, as crianças que fosse sorteadas randomicamente para o grupo controle entrassem em uma lista de espera e que tivessem a possibilidade de garantia de vaga para o ano seguinte; porém, tal procedimento não é aceitável tão pouco ético frente a estrutura do Guri. Em suma, escolhemos este modo de estudo, uma vez que seria impossível, por questões éticas inerentes ao processo democrático de inscrição no Programa[5], conduzir um estudo randomizado, que é tipo como o desenho padrão-ouro (em inglês, *gold-standard*) dentro da bioestatística clássica no processo de inferência causal (Boutron, Altman, Moher, Schulz & Ravaud, 2017; Greenland & Mansournia, 2015).

[5] O Programa não realiza processo seletivo. Embora exista uma lista de espera, que poderia funcionar como grupo controle no estudo randomizado, não seria ético obrigar as crianças dessa lista a esperar um ano inteiro para poderem se matricular, uma vez que, devido às desistências, é possível obter-se a vaga durante o ano.

4.3 Instrumentos

4.3.1 Instrumentos de controle

Questionário de critérios de classificação econômica da Associação Brasileira de Empresas de Pesquisa – ABEP (2018). Criado para avaliar o nível socioeconômico baseado em amostras de domicílios, contém 9 itens que avaliam a) posse de bens de consumo duráveis; b) tipo de sistema de abastecimento de água e de pavimentação na rua; c) número de residentes no domicílio; d) composição familiar; e) Grau de instrução do chefe da família. O resultado do questionário é uma medida estratificada em 5 classes socioeconômicas, sendo elas: A (subdividida em A1 e A2), B (também subdividida em B1 e B2), C, D e E.

Matrizes Progressivas Coloridas de Raven (CPM) – Escala Especial (Raven *et al.*, 2003): instrumento utilizado para avaliar a inteligência não-verbal, o raciocínio analógico e habilidade edutiva de crianças de 5 a 11 anos. É um dos instrumentos mais utilizados para a avaliação da inteligência não-verbal em crianças e adultos (Lúcio *et al.*, 2017; Pasquali, Wechsler, & Bensusan, 2002). Possui 36 itens divididos em três séries com 12 itens cada, em ordem gradual de dificuldade: série A, Ab e B. Cada item da série é composto por uma figura com um pedaço faltando, sendo a tarefa da criança indicar a figura que completa a parte faltante dentro das 6 opções de respostas apresentadas. O escore máximo na tarefa é de 36 pontos e a classificação da inteligência dada em percentis. O participante que apresentar Percentil 5 (classificação V) está em risco para dificuldades intelectuais e, portanto, será excluído da amostra.

4.3.2 Instrumentos de teste

Questionário de Capacidades e Dificuldades (SDQ) – O *Strengths and Difficulties Questionnaire* é uma medida validada, traduzida e adaptada culturalmente para mais de 15 línguas, com referências que nos auxiliam a ter parâmetros de aferição padronizados internacionalmente. O questionário foi desenvolvido originalmente por Goodman (1997), sendo a versão mais recente de Goodman e Goodman (2011), e tem por objetivo fornecer uma triagem de comportamentos, emoções e relacionamentos de crianças e adolescentes. Possui validação e adaptação para o português brasileiro (Stivanin, Scheuer, & Assumpção, 2008; Woerner *et al.*, 2004). O questionário apresenta duas versões, que podem ser respondidas para pais ou professores (os mesmos itens, adaptados ao respondente). São 25 afirmações que devem ser respondidas em uma escala tipo Likert de 3 pontos, composta das opções: falso, mais ou menos verdadeiro e verdadeiro (por exemplo: "é gentil com crianças mais novas?"). Os 25 itens estão organizados em 5 dimensões (sintomas emocionais, de conduta, hiperatividade, problemas de relacionamento e comportamento pró-social). Alguns itens são invertidos quanto à escala (p. ex., na seção de hiperatividade, se for respondido como "falso" à pergunta "pensa antes de agir", pontua-se com 2 pontos; mas se for falso "Está constantemente irrequieto ou agitado", pontua-se com zero ponto. Isso implica que, quanto maior a pontuação, maiores as dificuldades. De forma semelhante, no caso das forças (sociabilidade), aumenta-se conforme incremento dos escores. O resultado de cada escala pode ser avaliado se pelo menos 3 itens forem pontuados. A pontuação de 0 a 40 é dada para os itens de dificuldade (os itens de sociabilidade são pontuados de 0 a 10). Há uma segunda parte contendo informações de impacto, ou avaliam o estresse e prejuízo geral, que deve ser respondida caso o pai ou professor responda afirmativamente à primeira questão (se

responder negativamente, pontua-se com zero e interrompe-se a aplicação). Se o suplemento de impacto apresentar resultado de 0 é considerado normal, 1 limítrofe e 2 anormal.

Bateria psicológica para a avaliação da atenção (BPA, Rueda, 2013) – trata-se de um conjunto de subtestes que avaliam três domínios da atenção: atenção concentrada (capacidade de focalizar, selecionar e manter a atenção em estímulos alvo e ignorar estímulos concorrentes); atenção dividida (capacidade de prestar atenção a mais de um estímulo ao mesmo tempo, respondendo às diferentes necessidades do ambiente); e atenção sustentada (capacidade de alternar a atenção, ora para um estímulo, ora para outro). O teste apresenta estudos de validade e precisão e apresenta normas para pessoas entre 6 e 82 anos de idade.

Subteste Dígitos do WISC-IV (Wechsler, 2013): avalia a memória operacional verbal e a memória de trabalho verbal. Conjunto de números são apresentados para as crianças em ordem crescente de tamanho (2 a 8 sequências) e as crianças devem repetir na mesma ordem (dígitos direto) ou na ordem inversa (dígitos inverso) em que os números foram apresentados. Cada item apresenta duas tentativas, sendo que o subteste é interrompido quando a criança erra duas tentativas do mesmo item. O escore em cada tentativa é de um ponto. Desse modo, o escore máximo em cada subdivisão do subteste (direta ou inversa) é de 16 pontos, totalizando um escore total no subteste de 36 pontos.

Questionário de Comportamentos Agressivos e Reativos entre Pares (Q-CARP) - questionário traduzido e adaptado para o português brasileiro por Borsa e Bandeira (2014). O questionário contém ao todo 20 perguntas realizadas pelo aplicador à criança, envolvendo duas escalas distintas: a Escala de Comportamentos Agressivos – ECA, composta por cinco itens que avaliam agressividade física e verbal (p. ex., chutar ou dar um tapa) e três itens de controle que não são pontuados e servem de controle (p. ex., contar piadas). Esses itens referem-se à frequência de comportamentos e são perguntados como "quantas vezes acontece de você....", sendo que cada item apresenta uma escala tipo Likert de 4 pontos (todos os dias, às vezes, poucas vezes e nunca). A Escala de Reação à Agressão - ERA consiste em 12 frases que representam diferentes formas comumente relatadas por crianças para reagir à agressão de seus pares. A pergunta que se faz a estes itens é "quando um colega seu" seguida das asserções, que podem sinalizar reações agressivas (ex.: bate ou empurra você, você bate no seu colega?) ou outras formas de reação, como a busca por apoio (ex.: bate ou empurra você, você conta para a professora?) e reações emocionais internalizadas (ex.: bate ou empurra você, você chora e ficar emburrado?).

4.4 Procedimento de coleta de dados

Entre as crianças de 6 e 7 anos que fazem hoje duas horas de aula no Programa Guri Santa Marcelina, contabilizam-se 653 matriculadas em todos os polos. O Programa atua em 46 polos, cada um com características e peculiaridades distintas, informações que serão ajustadas analiticamente para que seja possível verificar diferenças na influência de efeito socioeconômico dos locais em que se encontram os polos do Programa.

Para prever a logística de aplicação dos testes e exames de imagem, será realizado um teste piloto de agosto a dezembro de 2019. Serão abordados 50 pais/responsáveis de crianças frequentadoras do projeto Guri para responderem ao SDQ de modo que se possa estimar a prevalência de crianças que participam do projeto e que apresentarão os pontos de corte para dificuldades apresentados no questionário SDQ. Os participantes que apresentarem os

referidos pontos de corte realizarão as avaliações individuais previstas (testes psicológicos) de modo a se verificar o tempo necessário para a aplicação dos instrumentos e se haverá necessidade de ajustes dos pontos de corte (p. ex., utilizar limítrofe ou anormal). Além disso, servirá para predizer a proporção esperada de crianças que deverão ser excluídas por desempenho intelectual abaixo do esperado (o que repercutirá em provável aumento do número de crianças sorteadas para compor o GE para as avaliações individuais de modo a obter o N amostral de 50 participantes).

O estudo principal será feito prospectivamente ao longo do ano escolar de 2020, ano em que serão levantados os dados de dois grupos: 1) o grupo interno, formado por 50 crianças de 6 e 7 anos expostas ao Programa; e 2) o grupo de controle, formado por 100 crianças da mesma faixa etária e bairro que não frequentam – nem nunca frequentaram – o Programa.[6] Os grupos serão avaliados em dois momentos distintos para realização dos instrumentos de triagem e para os testes cognitivos/emocionais: antes do início do ano letivo (triagem e pré-teste) e no final do ano letivo (pós-teste). Desse modo, a seguir, descrevem-se os procedimentos para a composição dos grupos (tratamento e controle) e para as avaliações.

No dia da matrícula das crianças no projeto Guri, os pais/responsáveis serão informados sobre a possibilidade de participação de seus filhos na pesquisa e serão convidados a assinar o Termo de Consentimento Livre e Esclarecido (TCLE). Após esta assinatura, serão convidados a responder ao questionário SDQ - versão para pais e o questionário da ABEP (2018) para avaliação do nível socioeconômico. Este procedimento constitui a fase de triagem e tem por objetivo identificar as crianças que frequentarão o Guri no ano de 2020 e que apresentam problemas nas cinco áreas avaliadas pelo SDQ (a saber, sintomas emocionais, problemas de conduta, hiperatividade, problemas de relacionamento). De posse dos resultados da triagem, serão aleatoriamente selecionadas 50 crianças[7] para a participação nas testagens individuais da pesquisa. Com a seleção das crianças que irão compor o grupo experimental (GE), serão identificadas as escolas que elas frequentam regularmente de modo a compor o grupo de controle (GC). Após autorização da direção das escolas para participação na pesquisa, os pais das crianças de mesmo sexo e que cursam a mesma turma de cada criança do GE selecionada serão convidados a participar da pesquisa e receberão o TCLE junto ao SDQ e ao questionário da ABEP. Os pais que retornarem o TCLE assinado terão o SDQ corrigido e os participantes que apresentarem os mesmos pontos de corte das crianças do grupo experimental serão sorteados para compor o GC.

Após a composição dos grupos, as crianças deverão partir para as avaliações individuais (pré-teste), em que serão avaliadas a inteligência não-verbal (teste de Raven) (Raven *et al*., 2003).

---

[6] O desbalanceamento 1:2 foi feito propositalmente para auxiliar a estimação dos potenciais efeitos da intervenção no processo de análise estatística. Os parâmetros para o cálculo amostral foram: teste-bicaudal de distribuição-t para duas amostras independentes com poder (1-erro tipo II) de 80%, nível de significância de 0,015 [dados que serão 4 medidas comportamentais principais de natureza contínua como desfecho], alocação de 1:2. Esse design permite identificar efeitos de magnitude moderada Cohen´s d=0,58 (Cohen, 1988). Ou seja, seríamos capazes de identificar diferenças entre os dois grupos, caso essas existam, se essas forem moderadas. Para maiores detalhes sobre as fórmulas e tamanho de efeito, ver Cohen (1988), Julious (2010), Machin et al. (2009), Ryan (2013).

[7] Este número poderá variar para cima, conforme resultados do estudo piloto para avaliação da inteligência.

As avaliações de neuroimagem serão organizadas em cronograma específico, uma vez que requer o deslocamento dos participantes até o local das avaliações. Para as avaliações da neuroimagem, serão randomizadas 50 crianças do grupo de controle para serem encaminhadas à realização de ressonância magnética (MRI), além das 50 que serão expostas ao Programa[8], sob supervisão da pesquisadora Profa. Dra. Andrea Jackowski do Departamento de Psiquiatria da Unifesp, colaboradora deste projeto.

Após o término do ano letivo, as crianças do GC e do GE serão reavaliadas em todas as tarefas individuais, incluindo exames de imagem, de modo a investigar o efeito do tratamento nas variáveis dependentes.

4.5. Coleta de exames de imagem

Todas as crianças passaram por um protocolo de dessensibilização com uma equipe de psicólogos antes de realizar o exame, a fim de aumentar a aceitação e diminuir o movimento durante o exame, uma vez que todo o protocolo de aquisição das imagens tem a duração de 20 minutos.

As imagens serão adquiridas no Departamento de Diagnóstico por Imagem (UNIFESP) em um aparelho de 3 Tesla. Todas as imagens serão analisadas na Universidade Federal de São Paulo (UNIFESP), no Laboratório Interdisciplinar de Neurociências Clínicas (LiNC-UNIFESP), coordenado pela Prof. Dra. Andrea Jackowski.

O protocolo de aquisição de imagem inclui as seguintes sequências:

- Uma série exploratória de imagens sagitais (nove a onze cortes de 5 mm com espaçamento de 1 mm).
- Neuroimagem estrutural: Serão adquiridas imagens ponderadas em T1 com os seguintes parâmetros de aquisição: MPRAGE; TR: 6.8; TE: 3.2; espessura do corte: 1.2; Matrix 256 x 240; ângulo de nutação: 9; tamanho do voxel: 1x1x1.2; número de cortes: 150.
- Resting-state fMRI (Ressonância magnética funcional em estado de repouso): Durante a aquisição dessa sequência, será solicitado que os sujeitos olhem para um ponto fixo sem realizar nenhuma tarefa específica. As imagens serão adquiridas com o seguinte padrão de aquisição: EPI; TR: 2000; TE 30; tamanho do voxel: 3x3x3; matriz de aquisição = 80x80 número de cortes: 38
- Imagens dos tensores de difusão (DTI): Serão adquiridas imagens spin-echo single-shot echo planar imaging (EPI) com os seguintes parâmetros: TR 5800 ms; TE: 77 ms; matrix: 128 x 128; FOV: 256 x 256 mm; in-plane resolution 2 mm; espessura do crote: 3 mm, 15 direções, e valor de b=0/1000.

4.6. Processamento e Análise das Imagens

As imagens de RM serão processadas utilizando diferentes plataformas de acordo com a modalidade de imagem em questão. As imagens T1, serão processadas utilizando o software *FreeSurfer* , que será descrito em detalhes nas próximas subseções.

---

[8] O Programa Guri Santa Marcelina mantém um acordo de cooperação técnica com a Secretaria Municipal de Educação, o que facilitará o acesso ao grupo de controle por meio das escolas localizadas próximas aos polos em que o Programa atua.

4.6.1. Medidas estruturais

Medidas de espessura cortical, índice de girificação local e volume das estruturas corticais e subcorticais serão calculados utilizando o software *FreeSurfer* (surfer.nmr.mgh.harvard.edu) (Fischl et al., 1999; Dale et al., 1999]). O *FreeSurfer* é um conjunto de ferramentas para pós-processamento e análise de dados de RM que se baseia em algoritmos de *watershed* e modelos de deformação de superfície. O *FreeSurfer* segmenta, de forma automatizada, estruturas corticais e subcorticais fornecendo medidas regionais de volume por compartimento (substância cinzenta e branca) bem como medidas de espessura cortical regional. Para esta fase do projeto será utilizada o procedimento para estudos longitudinais, que aumenta o poder estatístico e reprodutibilidade dos dados. Uma imagem média e um template específico para cada indivíduo será gerada, utilizando um registro inverso. Essa abordagem permite um aumento significativo na precisão enquanto mantém a habilidade de detectar alterações anatômicas [Reuter et al., 2012]. Para as análises de espessura cortical, utilizaremos o pacote *SurfStat* (http://www.math.mcgill.ca/keith/surfstat) para *Matlab*. Este software permite a análise de dados longitudinais utilizando modelos de efeitos mistos. Esse procedimento permite considerar indivíduos com intervalo entre scans diferentes e a dependência de dados para o mesmo indivíduo. As medidas de volume, média de espessura, área e curvatura de cada região, gerados durante a etapa de segmentação e parcelamento, serão exportados e utilizados em análises por região de interesse (ROI), utilizando o pacote estatístico SPSS (Statistical Package for Social Sciences, versão 20.0, Chicago IL, USA).

4.6.2. Resting-state fMRI

As imagens de ressonância magnética funcional (fMRI) serão pré-processadas utilizando rotinas do software FSL (http://fsl.fmrib.ox.ac.uk/fsl) e AFNI (afni.nimh.nih.gov), encapsuladas em scripts baseados na linguagem C-shell. As etapas de pré-processamento incluem: correção por movimento da cabeça, remoção do crânio e spikes, suavização espacial utilizando um kernel Gaussiano (FWHM=8mm), filtragem temporal passa-banda (0.01-0.1Hz) com detrend linear e quadrático, realinhamento das imagens funcionais com as imagens estruturais, onde as últimas são normalizadas espacialmente para o template MNI152 em um sistema de coordenadas padrão, segmentação das imagens estruturais em substância branca, cinzenta e liquor, extração do sinal BOLD médios das áreas segmentadas, filtragem do sinal BOLD de cada região removendo efeitos de flutuações do líquor, substância branca, sinal global, e dos seis parâmetros de movimento (rotação e translação) utilizando uma regressão linear múltipla.

A inferência de conectividade funcional por meio do processamento dos dados de fMRI pode ser realizada de duas maneiras: mapeamento a partir de uma região seed ou análise de regiões de interesse (ROI).O mapeamento da conectividade funcional via seed-voxel (consiste nas seguintes etapas: 1) Pré-processamento dos dados; Passo 2) Definição de um região seed; Passo 3) Cálculo da correlação (2 a 2) entre o sinal BOLD da região seed e cada um dos voxels da imagem, utilizando o coeficiente de Correlação de Pearson ou Spearman; Passo 4) Conversão do coeficiente de correlação em uma estatística Z, utilizando a transformada Z de Fisher; Passo 5) Limiarização do mapa considerando-se um determinado nível de significância (em geral 5%, corrigindo-se por comparações múltiplas utilizando-se o método FDR ou FWE).

Assim, utilizando-se a metodologia descrita acima, é possível identificar quais regiões do encéfalo estão funcionalmente conectadas à região seed. Em termos intuitivos, busca-se encontrar regiões cuja atividade espontânea (em um protocolo resting-state) está associada à atividade da região seed. Estes mapas de conectividade podem posteriormente serem utilizados como características ou variáveis de entrada dos métodos de aprendizado de máquina e reconhecimento de padrões. Uma segunda abordagem complementar ao mapeamento de conectividade funcional baseado na definição à priori de regiões de interesse (ROI; McIntosh, 1999). Uma ROI é definida por um conjunto de voxels espacialmente conectados, representando uma determinada região cerebral (ex: precuneus, porção dorsal do cíngulo anterior, etc). A média do sinal BOLD entre cada um dos voxels dentro da ROI é extraída e considerada como o sinal representativo da região. Esta abordagem pode ser considerada como uma forma de redução da alta dimensionalidade dos dados, pois as imagens são compostas de milhares de voxels. A análise da conectividade funcional é realizada considerando-se um conjunto de ROIs. Dessa forma, é possível calcular o coeficiente de correlação (2 a 2 ou parcial) entre os sinais das ROIs. A definição das ROIs pode ser realizada focando-se em um determinado circuito ou no cérebro todo utilizando-se um atlas. Para a abordagem exploratória utilizando-se regiões distribuídas no cérebro todo, pode-se usar alguns atlas pré-definidos.

4.6.3. Análise de Difusão

O pós-processamento das imagens ponderadas por difusão, resultando nas imagens de tensor de difusão (DTI), será realizado usando a plataforma FSL (software livre), versão 4.1.9 (Smith et al., 2006) de acordo com os seguintes passos:

· Correção das *eddy currents*, por meio do pacote de difusão (FDT) disponível na biblioteca de software FMRIB Library (Smith et al., 2006);

· Extração da caixa craniana por meio da ferramenta (BET) (Smith, 2006) com um fator de extração variável;

Por meio da ferramenta FDT da plataforma FSL e com base nos tensores, serão construídos os seguintes mapas: de anisotropia fracionada (FA), cujos valores normalizados variam entre 0 e 1, serão inspecionados para qualquer artefato ou movimento residual, de difusividade média (MD), e dos autovalores λ1, λ2 e λ3. Dependendo do tipo de estudo, se restringirá ao uso dos mapas de FA ou se estenderá ao uso dos demais mapas.

Os mapas de FA serão processados com o *TBSS* (*Tract Based Spatial Statistics)* seguindo o *pipeline* proposto por (Smith et al., 2006). Como a amostra é composta por crianças, considera-se mais adequado realizar o alinhamento sujeito a sujeito, a fim de se encontrar o indivíduo mais representativo da amostra. A imagem alvo selecionada sofrerá uma transformação linear seguida de uma translação (transformação afim) no espaço padrão MNI152 e cada imagem do estudo será transformada no espaço 1x1x1 do MNI152 combinando a transformação não linear de todas as imagens à imagem alvo e, finalmente, a transformação afim do alvo ao espaço MNI152. A média de todas estas imagens FA alinhadas resultará num único arquivo 4D com a imagem média da anisotropia fracional. Com base neste arquivo, o esqueleto médio do grupo será derivado, e os tratos mais relevantes de cada sujeito

espacialmente normalizado serão projetados neste esqueleto. Será aplicado o limiar recomendado de 0,2. Com base esqueleto médio (arquivo 4D) gerado pelo TBSS, será realizada uma extração automatizada de regiões de interesse (ROI), baseada nos 20 tratos do atlas JHU (John Hopkins University) de tractografia de substância branca (Wakana et al., 2007; Hua et al., 2008). Para extrair a ROI de cada sujeito, utilizaremos o programa *R-Project for Statistical Computing* (versão 3.0.2). Desta forma, o valor médio para cada um dos parâmetros separadamente (FA, MD, difusividades axial e radial) será então calculado baseado nos voxels pertencentes ao esqueleto médio. Os valores obtidos serão extraídos e processados em análise estatística apropriada ao desenho de cada estudo utilizando o SPSS (Statistical Package for Social Sciences, versão 20.0, Chicago IL, USA)

4.7 Considerações metodológicas e éticas

Uma vez que nossa hipótese é a de que a interferência do Programa possa ser um preditor de mudanças no prognóstico de habilidades cognitivas, sociais, e em estruturas cerebrais como desfecho, frisa-se aqui a importância na observação, análise e interpretação desses dados para que se possa, à luz das pesquisas que têm mostrado os efeitos das desigualdades sociais no cérebro e na saúde de crianças expostas a situações de risco, fornecer uma leitura que permita apresentar à sociedade e aos responsáveis pelos projetos de políticas públicas meios de diagnosticar os benefícios latentes de suas ações face aos malefícios da pobreza.

É importante salientar que se trata de projeto que, embora haja vários projetos sociais de educação musical (Ação Social pela Música do Brasil – ASMB em Chapéu Mangueira, Alemão, Macacos, Cidade de Deus, Piraí, João Pessoa e Rondônia, Neojibá, na Bahia, Projeto Guri, no interior e litoral paulista, Instituto Baccarelli, formalizado em 1996 na cidade de São Paulo, e Guri Santa Marcelina na Grande São Paulo), inexistem estudos quantitativos ou que tenham se utilizado de exames de imagem para medir o prognóstico que a exposição a programas sociais com foco em educação musical apresenta como desfecho no Brasil.

5. Forma de Análise dos Dados

Os dados serão inseridos pelos assistentes sociais e/ou bolsistas/monitores no banco de dados *online SurveyMonkey*, que gera automaticamente arquivos em Excel e gráficos, para posteriormente serem analisados estatisticamente pelo programa Mplus 8 e Stata versão 14.

Para a análise, serão reportados valores de média, desvio-padrão, mínimo e máximo para cada tarefa utilizada para a amostra geral e por grupo, no pré-teste e no pós-teste, tal como recomendado pelo CONSORT, especialmente considerando a extensão para estudos não farmacológicos (Boutron *et al*., 2017).

Para as estatísticas inferenciais, i.e., para verificar a generalização dos potenciais efeitos da intervenção versus o grupo de controle, será usada a técnica *Inverse Probability Weighting*. Essa técnica é adequada para diferentes cenários, como, por exemplo, estudos com dados perdidos nos diferentes desfechos (*missing data*, em inglês), *surveys* em que os sujeitos não foram obtidos por meio de processo randômico e também para estudos não-randomizados comparando duas intervenções (Mansournia & Altman, 2016). Resumidamente,

a técnica estatística estima o efeito causal da amostra e remove possíveis confundidores[9], baseando-se em um modelo de regressão logística que estima a probabilidade de exposição de uma determinada pessoa e usa a probabilidade prevista como um peso em análises subsequentes (para maiores informações ver Cattaneo, 2010; Cerulli, 2014, 2015, Wooldridge, 2010).

O objetivo é avaliar a exposição dicotômica de interesse (participar do projeto Guri *versus* não participar do projeto Guri), assumindo que a exposição ao projeto resultará em alterações pelo tipo causal no grupo exposto. Para falar em efeitos causais sem um processo randômico, se faz necessário estruturar um gráfico acíclico direcionado (DAG, Direct Acyclic Graph). A partir do DAG, que contém embutidas equações estruturais não paramétricas, será possível verificar se a quantidade alvo de interesse é identificável a partir das variáveis coletadas pelo pesquisador, aplicando-se, por exemplo, o critério da porta de trás (*back door criterion*) de Pearl (2009a).

Os confundidores serão controlados por correspondência (*matching*), tomando por base o modelo que prevê níveis similares de confundidores entre os grupos de exposição e o de não exposição.

Além dessa análise central, serão realizadas regressões lineares para avaliar as respostas da escala comportamental SDQ (Goodman & Goodman, 2011), do questionário econômico ABEP (2018), do Questionário de Comportamentos Agressivos e Reativos entre Pares (Q-CARP; Borsa & Bandeira, 2014), memória de trabalho verbal (WISC-IV), atenção concentrada, dividida e sustentada (Rueda, 2013) e o teste de Matrizes Coloridas de Raven (Raven *et al.*, 2003); cujas respostas serão colocadas como preditores do desfecho de exposição ao projeto.

6. Equipe

Responsável: Profa. Dra. Graziela Bortz

Pesquisa anterior com fomento Fapesp - Auxílio Regular: 2014/03322-1 (Percepção melódica produtiva e receptiva: avaliação e validação de critérios)

Resultado: Fapesp auxílio publicação 2018/09500-0

Bortz, G., Germano, N. G., Cogo-Moreira, H. (2018). (Dis)agreement on sight-singing assessment of undergraduate musicians. *Frontiers in Psychology (Cognition).*

Colaboradores:

Profa. Dra. Andrea Jackowski (Unifesp)
Profa. Dra. Patrícia Silva Lúcio (UEL)
Prof. Dr. Hugo Cogo-Moreira (Freie Universität Berlin/Unifesp)
Profa. Dra. Nayana Di Giuseppe Germano (UFSM)
Profa. Beatriz Ilari (USC, Thornton)

[9] Confundidores são variáveis que podem influenciar tanto o preditor quanto o desfecho, ocasionando resultados espúrios. É necessário identificar os possíveis confundidores e controlá-los para resultados mais confiáveis.

7. Implantação pela Instituição Parceira

A Instituição Parceira, Associação de Cultura, Educação e Assistência Social Santa Marcelina, cujo convênio com a Universidade Estadual Paulista (Unesp) foi celebrado em 5 de setembro de 2018, oferece como contrapartida para a pesquisa a colaboração de assistentes sociais, técnicos administrativos, estagiários, acesso aos polos nos quais o Programa Guri Santa Marcelina opera, além de colaborar com a interlocução com a Secretaria Municipal de Educação, facilitando o acesso aos estudantes do grupo de controle. Embora não esteja prevista contrapartida financeira, uma vez que depende de verbas governamentais, a Associação oferece sua infraestrutura institucional.

Os assistentes sociais do Programa Guri Santa Marcelina serão atores cruciais na implantação do trabalho junto às comunidades localizadas na Grande São Paulo, uma vez que são profissionais experientes e que gozam da confiança dessas comunidades. Esses profissionais e estagiários da instituição parceira serão treinados junto aos bolsistas técnicos para aplicar os questionários e escalas, instrumentos esses que poderão servir de acompanhamento em larga escala após a conclusão da pesquisa aqui proposta, na fase de implantação dos resultados, podendo dar continuidade ao levantamento de dados e aplicação dos instrumentos capazes de fornecer informações e pistas para o acompanhamento constante da evolução e da intervenção do Programa, seja em cada polo, seja no Programa como um todo.

8. Cronograma

**Ano**	**Jan-Mar**	**Abr-Mai**	**Jun-Jul**	**Ago**	**Set-Out**	**Nov**	**Dez**
**2019**				**PILOTO: MRI, SDQ, ABEP, Raven e MT; BPA; Q-CARP;**		Avaliação de estratégias e logística	
**2020**	**SDQ, ABEP, Raven, MT; BPA; Q-CARP e** MRI	Survey Monkey	Relatório parcial/ Prestação de contas	**SDQ, ABEP, Raven, MT; BPA; Q-CARP e** MRI	Survey Monkey	**SDQ, ABEP, Raven, MT; BPA; Q-CARP e** MRI	Survey Monkey
**2021**	Análise estatística<br>Análise MRI<br><br>Produção de artigos<br>Submissão dos resultados em periódicos internacionais		Relatório Final / Prestação de contas		Implantação Instituição Parceira		
**2022**	Implantação Instituição Parceira						

9. Referências

Alemán, X., Duryea, S., Guerra, N. G., McEwan, P., Muñoz, J. R., Stampini, M., & Williamson, A. A. (2016). The effects of musical training on child development: a randomized trial of El Sistema in Venezuela. *Prevention Science, 18*, 865-878.

Associação Brasileira de Empresas de Pesquisa - ABEP. (2018). Critério de classificação econômica Brasil. Disponível em: http://www.abep.org/criterio-brasil Acesso em 31 de maio, 2018.

Blair, C. (2010). Stress and the development of self-regulation in context. *Child Development Perspectives, 4*(3), 181-188.

Blair, C., Berry, D., Mills-Koonce, R., Granger, D., & the FLP Investigators (2013). Cumulative effects of early poverty on cortisol in young children: moderation by autonomic nervous system activity stress and poverty. *Psychoneuroendocrinology 38*(11), 2666–2675.

Borsa, J. C., & Bandeira, D. R. (2014). Adaptação transcultural do questionário de comportamentos agressivos e reativos entre pares no Brasil. *PsicoUSF. 19*(2), 287-296.

Boutron, I. Altman, D. G., Moher, D., Schulz, K. F., & Ravaud, P. D. J. C. (2017) CONSORT Statement for Randomized Trials of Nonpharmacologic Treatments: A 2017 Update and a CONSORT Extension for Nonpharmacologic Trial Abstracts. *Annals of Internal Medicine*. *American College of Physicians, 167*(1):40. doi: 10.7326/M17-0046.

Cattaneo, M. D. (2010). Efficient semiparametric estimation of multi-valued treatment effects under ignorability. *Journal of Econometrics* 155: 138–154.

Cerulli, G. (2014). Ivtreatreg: A command for fitting binary treatment models with heterogeneous response to treatment and unobservable selection. *Stata Journal* 14: 453–480.

Cerulli, G. (2015). *Econometric Evaluation of Socio-Economic Programs: Theory and Applications*. Berlin: Springer.

Cohen, J. (1988). *Statistical Power Analysis for the Behavioral Sciences*. Hillsdale, NJ: Lawrence Erlbaum Associates.

Corwin, E. J., Guo, Y., Pajer, K., Lowe, N., McCarthy, D., Schmiege, S., Weber, M., Pace, T., & Stafford, B. (2013). Immune dysregulation and glucocorticoid resistance in minority and low income pregnant women. *Psychoneuroendocrinology 38*(9), 1786–1796.

Dale, A. M., Fischl, B., Sereno, M. I. (1999). Cortical surface-based analysis. I. Segmentation and surface reconstruction. *NeuroImage*, *9*(2):179-194.

Fink, A., Grabner, R. H., Benedek, M., & Neubauer, A. C. (2006). Divergent thinking training is related to frontal electroencephalogram alpha synchronization. *European Journal of Neuroscience*, *23,* 2241–2246.

Goodman, R. (1997). The Strengths and Difficulties Questionnaire: A research note. *Journal of Child Psychology and Psychiatry, 38*, 581-586.

Goodman, A., & Goodman, R. (2011). Population mean score predict child mental disorder rates: validating SDQ prevalence estimators in Britain Stress. *Journal of Child Psychology and Psychiatry, 52*, 100-108.

Greenland, S., & Mansournia, M. A. (2015). Limitations of individual causal models, causal graphs, and ignorability assumptions, as illustrated by random confounding and design unfaithfulness. *European Journal of Epidemiology,30*(10), 1101–1110.

Habibi, A., Cahn, B. R., Damasio, A., & Damasio, H. (2016). Neural correlates of accelerated auditory processing in children engaged in music training. *Developmental Cognitive Neuroscience, 21*, 1-14.

Holochwost, S. J., Propper, C. B., Wolf, D. P., Willoughby, M. T., Fisher, K. R., Kolacz, J., Volpe, V. V., & Jaffee, S. R. (2017b). *Psychology of Aesthetics, Creativity, and the Arts, 11*, 147-166.

Holochwost, S. J., Wolf, D. P., & Bose, J. H. (2017a). Building strengths, buffering risk: evaluating the effects of El Sistema-inspired music programs in the United States. Disponível em: http://wolfbrown.com/images/books_reports/Building_Strengths_Buffering_Risk.pdf Acesso em 17/02/2018.

Hua, K., Zhang, J., Wakana, .S, Jiang, H., Li, X., Reich, D. S., Calabresi, P. A., Pekar, J. J., van Zijl, P. C., Mori, S. (2008). Tract probability maps in stereotaxic spaces: analyses of white matter anatomy and tract-specific quantification. *NeuroImage*, *39*(1):336-347.

Huron, D. (2001). Is Music an evolutionary adaptation? In I. Peretz & R. Zatorre (Eds.). *The Cognitive Neuroscience of Music* (pp. 57-75). Oxford: Oxford University Press.

Ilari, B. S., Keller, P., Damasio, H., & Habibi, A. (2016). The development of musical skills of underprivileged children over the course of 1 year: A study in the context of an El Sistema-inspired program. *Frontiers in Psychology*, 7.

Jelinek, L., Randjbar, S., Seifert, D., Kellner, M., & Moritz, S. (2009). The organization of autobiographical and nonautobiographical memory in posttraumatic Stress Disorder (PTSD). *Journal of Abnormal Psychology 118*(2), 288-298.

Julious, S. A. (2010). *Sample Sizes for Clinical Trials*. Boca Raton, FL: Chapman & Hall/CRC.

Juruena, M. F., Clearea, A. J., & Pariante, C. M. (2004). O eixo hipotálamo-pituitária-adrenal, a função dos receptores de glicocorticóides e sua importância na depressão. *Revista Brasileira de Psquiatria, 26*(3), 189-201.

Lent, Robert. (2010). *Cem Bilhões de Neurônios*. Rio de Janeiro: Atheneu.

Lúcio, P. S., Cogo-Moreira, H., Puglisi, M., Polanczyk, G. V., & Little, T. D. (2017). Psychometric Investigation of the Raven's Colored Progressive Matrices Test in a Sample of Preschool Children. *Assessment*. Advance online publication. doi:1073191117740205.

Machin, D., Campbell, M., Tan, B. T., Tan, S. H. (2009). *Sample Size Tables for Clinical Studies.* 3rd Edition. Hoboken, NJ: Wiley-Blackwell.

Mansournia, M. A, & Altman, D. G. (2016). Inverse Probability Weighting. *BMJ.* Research Methods & Reporting. doi: 10.1136/bmj.i189.

McEwen, B. S. (2001). Plasticity of the hippocampus: adaptation to chronic stress and allostatic load. *Annals of the New York Academy of Sciences*, 265-277.

McIntosh, A. R. (1999). Mapping cognition to the brain through neural interactions. *Memory*, *7*(5-6):523-548.

Milner, H. R. (2012). Beyond a test score: explaining opportunity gaps in educational practice. *Journal of Black Studies,*43(6), 693-718.

Ministerio del Poder Popular (MPP), Venezuela. (2018). *El Sistema.* Disponível em: http://fundamusical.org.ve/category/el-sistema/que-es-el-sistema/#.Woil8uZzLIU Acesso em 17/02/2018

Pasquali, L., Wechsler, S., & Bensusan, E. (2002). Matrizes Progressivas do Raven Infantil: um estudo de validação para o Brasil. *Avaliaçao Psicologica: Interamerican Journal of Psychological Assessment*, *1*(2), 95-110.

Pearl, J. (2009). Causal inference in statistics: An overview. *Statistics surveys*, *3*, 96-146.Pinker, S. (2015). *Como a Mente Funciona* (3ª ed., L. T. Motta, Trad.). São Paulo: Companhia das Letras. (Obra original publicada em 1997).

Raven, J., Raven, J. C. & Court, J. H. (2003). Matrizes Progressivas Coloridas de Raven: Manual. (Trad. e Adapt. J. J. de Paula, C. G. M. F. Schlottfeldt, L. F. M. Diniz, G. A. A. Mizuta). São Paulo, SP: Pearson, 2018.

Reuter, M., Schmansky, N. J., Rosas, H. D., Fischl, B. (2012). Within-subject template estimation for unbiased longitudinal image analysis. *NeuroImage*, *61*(4):1402-1418.

Rueda, F. J. M. (2013). *Coleção BPA - Bateria Psicológica de Atenção*. São Paulo: Vetor.

Secretaria da Cultura do Estado de São Paulo (SEC-SP) (2017). *Programa Guri Santa Marcelina.* Disponível em: http://gurisantamarcelina.org.br/estude-musica/sobre-o-programa/ acessado em 17/02/2018

Sergent, J., Zuck, E., Terriah, S., & MacDonald, B. (1992). Distributed neural network underlying musical sight-reading and keyboard performance. *Science, 257,*106-109.

Sheridan, M. A., Foxd, N. A., Zeanahe, Charles H., McLaughlinb, K. A., & Charles A. Nelson. (2012). Variation in neural development as a result of exposure to institutionalization early in childhood. *PNAS, 9*(23), 12927–12932.

Smith, S. M., Jenkinson, M., Johansen-Berg, H., Rueckert, D., Nichols, T. E., Mackay, C. E., Watkins, K. E, Ciccarelli, O., Cader, M. Z., Matthews, P. M. (2006). Tract-based spatial statistics: voxelwise analysis of multi-subject diffusion data. *NeuroImage*, *31*(4):1487-1505.

Springer, S., & Deutsch, G. (1998). *Cérebro esquerdo, cérebro direito*. (4ª ed., T. Yoshiura). São Paulo: Summus Editorial (obra original publicada em 1993).

Stephens, M. A. C., & Wand, G. (2012). Stress and the HPA axis role of glucocorticoids in alcohol dependence. *Alcohol Research: Current Reviews, 34*(4), 468-483.

Stivanin, L., Scheuer, C. I., & Assumpção Jr, F. B. (2008). SDQ (Strengths and Difficulties Questionnaire): identificação de características comportamentais de crianças leitoras. *Psicologia: Teoria e Pesquisa*, *24*(4), 407-413.

Trehub, S. E. (2001). Musical predispositions in infancy: an update. In I. Peretz & R. Zatorre (Eds). *The Cognitive Neuroscience of Music* (pp. 3-20). Oxford: Oxford University Press.

Wakana, S., Caprihan, A., Panzenboeck, M. M., Fallon, J. H., Perry, M., Gollub, R. L., Hua K., Zhang J., Jiang, H., Dubey, P. (2007). Reproducibility of quantitative tractography methods applied to cerebral white matter. *NeuroImage, 36*(3):630-644.

Wechsler, D. (2013). *Escala Weschsler de inteligência para crianças: WISC-IV. Manual Técnico.* Tradução do manual original Maria de Lourdes Duprat. (4. ed.). São Paulo: Casa do Psicólogo.

Weichselbaum, A., & Nunes, P. L. (2016). Contribuições do ensino da música em projetos sociais: depoimentos de egressos. *XVII Encontro Regional Sul da ABEM,* 1-14.

Woerner, W., Fleitlich-Bilyk, B., Martinussen, R., Fletcher, J., Cucchiaro, G., Dalgalarrondo, P., ... & Tannock, R. (2004). The Strengths and Difficulties Questionnaire overseas: evaluations and applications of the SDQ beyond Europe. *European child & adolescent psychiatry*, *13*(2), ii47-ii54.

Wooldridge, J. M. (2010). *Econometric Analysis of Cross Section and Panel Data*. 2nd ed. Cambridge, MA: MIT Press.

Zalewski, M., Lengua L. J., Kiff C. J., & Fisher P. A. (2012). Understanding the relation of low income to HPA-axis functioning in preschool children: cumulative family risk and parenting as pathways to disruptions in cortisol. *Child Psychiatry Human Development 43*(6), 924–942.
