# Supplementary material for: The impact of music education on children’s cognitive and socioemotional development: A quasi-experimental study in the Guri Program in Brazil
Source: PLoS One. 2025 Oct 16;20(10):e0314355. doi: 10.1371/journal.pone.0314355 (PMC12530532; doi:10.1371/journal.pone.0314355)
Supplement: Attachment — Submitted filename: renamed_4c490.docx [file pone.0314355.s004.docx]

# COMMENTS – Manuscript PONE-D-24-48345

Title: “Music education reduces emotional symptoms in children: A quasi-experimental study in the Guri Program in Brazil”

**Important note: This review pertains only to ‘statistical aspects’ of the study and so ‘clinical aspects’ [like medical importance, relevance of the study, ‘clinical significance and** **implication(s)’ of the whole study, etc.] are to be evaluated [should be assessed] separately/independently. Further please note that any ‘statistical review’ is generally done under the assumption that study specific methodological [as well as execution] issues are perfectly taken care of by the investigator(s). This review is not an exception to that and so does not cover clinical aspects {however, seldom comments are made only if those issues are intimately / scientifically related & intermingle with ‘statistical aspects’ of the study}. Agreed that** **‘statistical methods’ are used as just tools here, however, they are vital part of methodology [and so should be given due importance]. I look at the manuscript in/with statistical view point, other reviewer(s) look(s) at it with different angle so that in totality the review is very comprehensive. However, there should be efforts from authors side to improve (may be by taking clues from reviewer’s comments). Therefore, please do not limit the revision only (with respect) to comments made here.**

**COMMENTS:** I have different opinion/views/observations/concerns or rather questions regarding quite a few issues [including some very serious ones] which are given below:

I noted that your ABSTRACT is drafted alright (in my opinion), but is ‘assay type’. It is preferable [refer to item 1b of CONSORT checklist 2010: Structured summary of trial design, methods, results, and conclusions] to divide the ABSTRACT with small sections like ‘Objective(s)’, ‘Methods’, ‘Results’, ‘Conclusions’, etc. which is an accepted practice of most of the good/standard journals [including this one, though ‘The PLoS One Guidelines to Authors’ did not specify an Abstract format, it is desirable]. It will definitely be more informative then, I guess, whatever the article type may be {though Section headings may differ for different Article Types [example: Study Protocol]}. Moreover, your abstract is very short (only 188 words).

Note that the effect size {eta-squared from an analysis of variance (ANOVA) test} [A significant effect of the intervention in emotional symptoms in the music group as opposed to controls was found (*F*(1,89) = 4.562, *p* = 0.035, *η^2^* = 0.049), indicating benefits for the music group] is very-very small. Reference quoted in line 326 {13. Miller E, Cohen J. An Integrative Theory of Prefrontal Cortex Function. Annual review of neuroscience. 2001; 24: 167-202 is different } is diffent than one given below. How that indicates “benefits for the music group” is a serious question. The manuscript is on very valid & very interesting topic. However, in such studies/trials the effect size generally observed/expected to be small and so needs ‘large sample’ sizes (for both groups). Please refer to table-1 on page 157 of Jacob Cohen’s paper “A power primer” in Psychological Bulletin, 1992, vol.:112, pp 155-159 [which is a sort of summary of the excellent book by Cohen himself titled ‘Statistical power analysis for the behavioral sciences’, Academic Press, 1977, New York] for magnitude of various effect size indexes. How the ‘effect of the intervention in emotional symptoms’ measured (as one entity) is not described (most questionable)?

Inference “Children whose mothers had low levels of education benefited with gains between 0.70 and 0.95 standard deviations in both groups for divided attention, indicating a significant interaction with maternal education” is wonderful [least understood, more explanation required]. From where or how it is drawn is not understood (highly questionable)? This way of interpreting/expressing result(s) appears to be new to me. Kindly let me learn more/novel things {by giving details}.

Most objectional/questionable issue is regarding ‘sample size’. Footnote-4 of line 169 (we intended to test children aged between 6 and 7, but as it was difficult to achieve the minimum sample size desired in this group, the range was extended) shows that the formal ‘desired/required sample size’ estimation was done sometime. But surprisingly it is not discussed at all (anywhere) in the manuscript. You have not given/discussed ‘How the required minimum sample size for this study was determined’ which nevertheless is a very-very important question [one of the important items in CONSORT checklist, item 7a] for any type of study (clinical trial or else). This point needs to be discussed in adequate details {including assumptions made at the time of estimation, power (confidence/accuracy/precision in case of single-arm/group studies) of the study, software used, etc.}. One concludes that the sample size used in this study is haphazardly determined. {even for required sample size use the above reference [“A power primer” in Psychological Bulletin, 1992, vol.:112, pp 155-159]}

As you may (rather definitely) know that “all ‘Clinical Trials’ must follow CONSORT guidelines”. Since your article type is ‘Clinical Trial’, you are supposed to cover these items in the report. In addition, you may please refer to “ICH HARMONISED TRIPARTITE GUIDELINE STATISTICAL PRINCIPLES FOR CLINICAL TRIALS - E9” latest version of which is available on NET/WWW. Although you mentioned in the title itself [and also clarified later in lines 163-66: Due to ethical reasons related to the policies of enrollment in the Guri Program, it was not possible to randomize child participants into music and control groups. Therefore, we performed an intervention study based on a quasi-experimental design] that it ‘A quasi-experimental study’, the journal rightly classified this study as a clinical trial. Choice/selection of the ‘control’ group is doubtful regarding its correctness. Please note that non-random allocation does not make it a different (like observational) study and so you are supposed to follow “CONSORT guidelines”.

Though the measures/tools used are appropriate often times, most of them [example: statement in lines 187-88 After signing consent forms, parents were invited to complete the SDQ–parent version and the ABEP questionnaire] are likely to yield data that are in ‘ordinal’ level of measurement [and not in ratio level of measurement for sure {as the score two times higher does not indicate presence of that parameter/phenomenon as double (for example, a Visual Analogue Scales VAS score or say ‘depression’ score)}]. In such situation one has to inflate sample size by 10 to 20% and also then the application of suitable non-parametric (or distribution free) test(s) is/are indicated/advisable [even if distribution may be ‘Gaussian’ (also called ‘normal’)]. Agreed that there is/are no non-parametric test(s)/technique(s) available to be used as alternative in all situation(s), but should be used whenever/wherever they are available. Therefore, in short use suitable non-parametric test(s)/technique(s) while dealing with data that are in ‘ordinal’ level of measurement even if [despite that] the distribution may be ‘Gaussian’. Testing ‘normality’ in sample [by using any normality test(s)} is not required/desired while dealing with data that are in ‘ordinal’ level of measurement [as most of the normality tests are not valid for ‘ordinal’ data].

In the ABSTRACT [The music group (n = 38, 5-9 years) was recruited from 10 centers (*polos*) distributed across the metropolitan area of São Paulo and the control group (n = 67) consisted of aged-matched children who attended public schools surrounding the *polos*.] as well as in lines 181-2 it is stated that “The control group consisted of aged-matched children who attended regular schools surrounding the Guri *polos*” but sample sizes of these two groups are different, therefore I am curious to know the method used here for matching. Please note that similar age group coverage does not mean ‘aged-matched’. What exactly you want to convey by saying in lines 315-6 “We paired the groups using pre-test data (before running the analyses, using data from all children who were paired).”. In my knowledge, the term is ‘age-matched’ and not ‘aged-matched’. Kindly check for the ‘English’ language. Agreed that English is not our mother tongue (definitely not mine, may or may not be yours but certainly not of many readers), however in any case, remember/ Kindly mind you [please excuse me for such a harsh comment/statement] that **this is a scientific/academic document and so all details should be clearly/correctly communicated (do not take reader’s for granted)**. You may take help of language professional expert, if needed.

Way used for matching (lines 288-290: “we managed the sample characteristics to match study groups. Sample matching was carried out based on the removal of children from both groups”) is also highly objectionable. Difference (large) in sample sizes of two groups [experimental/intervention group & control group] is also questionable. Method of imputation used [an expectation–maximization (EM) algorithm which is an iterative method to find (local) maximum likelihood or maximum a posteriori (MAP) estimates of parameters in statistical models, where the model depends on unobserved latent variables] is also questionable. Application of expectation–maximization (EM) algorithm as a method of imputation is not found on NET/WWW search {above quote about this method is after/by NET/WWW search only}.

When only two independent groups are to be compared {ex. Women and Men}, we use ‘t’ test for two independent groups [non-parametric equivalent to unpaired ‘t’ test is Mann-Whitney ‘U’ test] and not ANOVA ‘F’. Although ‘F’ and ‘t’ are mathematically related/equivalent [square of ‘t’ is exactly equal to ‘F’ if (mistakenly) calculated for two groups], logic/philosophy (and so underlying assumptions) behind their development and algorithms used for estimation of test statistic are different. They are applicable in different situations. I do not see any ‘repeated measures’ (lines 320-321: Inferential analyses are reported by ANOVAS with repeated measures). ‘Pre-Post’ differences could be delt by Mann-Whitney test on ‘change scores’ or by applying ANCOVAs. In fact, whole ‘Statistical analysis’ (including section described in lines 310 onwards) is of questionable value.

Limitation of this study highlighted in lines 484-5 “As limitations of this study, the absence of statistically significant interaction between the groups in some cases may be due to the low sample size and short intervention time” is appreciated but note that there are quite a few other limitations as well [important ones are ‘design’ of the study and way of analyses].

As pointed out in ‘important note’ above “This review pertains only to ‘statistical aspects’ of the study and so ‘clinical aspects’ should be assessed separately/independently [one should carefully consider/look at the clinical implications of the study]. In my opinion,

to rescue this article (which little difficult, but not impossible), large amount of re-vision (re-drafting) may be needed. However, please do not limit the revision only (with respect) to comments made here. More improvement is expected. Nevertheless, ‘how to handle/accommodate these suggestions?’ is questionable as the study is already conducted/complete. ‘Major revision’ is recommended [as the study is on very interesting topic and therefore, would like to give chance to authors for improvement of the manuscript].
