# Supplementary material for: The impact of music education on children’s cognitive and socioemotional development: A quasi-experimental study in the Guri Program in Brazil
Source: PLoS One. 2025 Oct 16;20(10):e0314355. doi: 10.1371/journal.pone.0314355 (PMC12530532; doi:10.1371/journal.pone.0314355)
Supplement: Attachment — Submitted filename: Response to reviewers.docx [file pone.0314355.s006.docx]

June 15, 2025

We are very grateful for the feedback and observations regarding the CONSORT guidelines. We realized that a different approach to reporting the study was necessary. Alongside this letter and the revised manuscript, we have carefully addressed all the questions and observations raised through the reviewers’ attentive reading of our work.

Because the revised manuscript underwent significant changes—particularly due to modifications in the statistical analysis—we took the liberty of reporting all the changes directly in the text, rather than responding point by point. All modifications are highlighted in red in the manuscript.

- In accordance with item 1b of the CONSORT 2010 checklist, we revised the structure of the Abstract.

- As noted by Reviewer 1, our study is best described as a quasi-experimental study, and the journal has correctly classified it as a clinical trial. Therefore, it must adhere to the CONSORT guidelines. We acknowledge a serious mistake in our sample matching and imputation methods. According to CONSORT, the complete dataset of the selected sample must be reported, and Little’s MCAR test is not the most appropriate choice for this type of data. Based on this observation, we returned to the protocol and revised our inclusion criteria.

- In the Methods section, we added a Design subsection to clarify the quasi-experimental nature of the study. In the Sample Recruitment section, we provided a more detailed explanation of the target sample (see page 8 onwards) and the exclusion process prior to sample assignment. We also added Footnote 4 to explain the recruitment criteria and amendments to the study protocol. Consequently, the flowchart (Fig. 1) was updated. All supplementary tables and Figure 1 have been modified, and supplementary tables as well as Figures 2 through 4 have been deleted.

- For the statistical analysis, multiple imputation was used instead of Little’s MCAR test. We conducted independent samples t-tests and chi-squared tests to examine baseline differences between the music group and the control group. Since SPSS does not provide pooled results for multiply imputed data, standard deviations, Cohen’s d, and chi-square statistics were manually calculated in Excel using Rubin’s rules [Rubin DB. Multiple imputation for nonresponse in surveys. Wiley; 1987].

- To test the intervention effect, generalized linear models (GLMs) were used instead of ANOVAs, as they are more appropriate for this type of data. Maternal education was included as a covariate due to baseline differences between the groups. Finally, Bonferroni’s correction was applied to adjust p-values for multiple comparisons.

- As a result, our data did not support significant differences between the groups in terms of behavioral problems, cognitive abilities, or aggression. The few observed effects point to developmental changes in both groups. For this reason, we also changed the title to: The impact of music education on children's cognitive and socioemotional development: A quasi-experimental study in the Guri Program in Brazil.

Although the results—and consequently their interpretation—have changed substantially, we still believe the paper makes a meaningful contribution to the literature on music education and its potential impact on behavioral characteristics. Few clinical trials have explored the relationship between music education, cognitive and socioemotional outcomes. The methodological changes introduced aim to improve the assessment of this relationship, and implications for future research are discussed.
