# Supplementary material for: The impact of music education on children’s cognitive and socioemotional development: A quasi-experimental study in the Guri Program in Brazil
Source: PLoS One. 2025 Oct 16;20(10):e0314355. doi: 10.1371/journal.pone.0314355 (PMC12530532; doi:10.1371/journal.pone.0314355)
Supplement: Attachment — Submitted filename: Response_to_reviewers_auresp_2.docx [file pone.0314355.s007.docx]

August 21st, 2025

Dear editor and reviewers,

We are immensely grateful for the careful reading and comments received, which certainly improved the quality of our manuscript. We would like to inform you that all the reviewers' suggestions were considered in this revision of the manuscript, as follows:

**Reviewer #1: COMMENTS: I note that most of the comments made on earlier draft are attended to the possible extend. However, the original over-all quality of the manuscript generally remains the same [as the study is already completed & I am not fully satisfied]. Now I recommend the acceptance of this manuscript for publication [only because the study (is on important topic and) has potential]. However, note the following:**

**Although all modifications are highlighted in red in the manuscript, reporting the changes directly in the text, makes the review process difficult and time consuming. Responding to comments point by point could have been very useful and so appreciated.**

We greatly appreciate your consideration in accepting the manuscript, even though you likely identified methodological or technical issues with the study's execution.

We regret that our response method made it difficult for you to understand the changes. Because the statistical analysis changed drastically from Round 1 to Round 2, and consequently the results and their interpretation, we chose to summarize the changes in the letter. We will consider this comment in future submissions, and we are grateful for it.

**Reviewer #2: I appreciate the authors’ thoughtful and extensive revisions to the manuscript. However, the revised statistical approach still does not appropriately test the study’s central hypothesis.**

**The current modeling strategy uses follow-up scores as the dependent variable while adjusting for baseline and maternal education. This design may control for baseline differences but does not directly test whether the groups change differently over time.**

**Without modeling the group × time interaction or using a repeated-measures framework, the analyses do not support valid inferences about the effectiveness of the music education intervention.**

**I strongly encourage the authors to reanalyze the data using a repeated-measures GLM that includes time, group, and their interaction. This is standard practice for evaluating intervention effects in longitudinal designs.**

We greatly appreciate your feedback. We have reorganized the database and modified the statistical analysis. We use Generalized Estimating Equations (GEE) instead of General Linear Models (GLM), following this recommendation. On pages 12-13, lines 295-302 (Statistical Analysis), we state:

The intervention effect was analyzed using Generalized Estimating Equations (GEE), an approach suitable for modeling longitudinal or repeated-measures data, as it considers the dependence of observations within individuals and groups. Respondents were specified as the within-subject factor, and assessment time (baseline and FUP) was modeled as the repeated measure. An exchangeable working correlation structure was assumed. Group and assessment time were included as fixed factors, with maternal education entered as a covariate. Main effects were reported, and the group × time interaction was tested to assess the intervention’s effectiveness.

Moreover, in the results section, we explained how to interpret the results accordingly (page 16, lines 344-359):

GEE models were used to verify the impact of musical intervention on the different variables of the study. For the SDQ analysis, difficulties (*i.e.*, hyperactivity, relationship problems, conduct problems, emotional symptoms, and difficulties in general) and prosocial behavior were considered as dependent variables. We considered the group (control, experimental) and time (baseline, FUP) as factors and maternal education as covariate. Due to the ordinal nature of this variable, dummy codes were created using the lowest level of the maternal education (illiterate) as the reference variable. The control group and baseline were used as references.

In the GEE analysis, the main effect of groups indicates whether the groups differ significantly on the target variable, regardless of time. Similarly, the main effect of time indicates whether scores on the target variable change (decrease or increase) regardless of group. These results are adjusted for maternal education. The effect of the intervention is reflected in the presence of a significant group × time interaction, which shows whether changes in the dependent variable (decrease or increase) differ between groups. We expect a greater reduction in symptoms or a greater increase in cognitive variables for the treatment group compared to the control group. Baseline and FUP comparisons are shown in Table 4.

Some results were changed with the correct analysis. In this version of the manuscript, no significant results were found, except for changes in developmental difficulties (total score) and alternating attention.

We appreciate the time you took to carefully review our work.
